# Impact of Diabetes on The Effects Of Sodium Glucose Co-Transporter-2 (SGLT2) Inhibitors on Kidney Outcomes: Collaborative Meta-Analysis of Large Placebo-Controlled Trials

**DOI:** 10.1016/S0140-6736(22)02074-8

**Published:** 2022-11-04

**Authors:** Writing Committee, Natalie Staplin, Richard Haynes, Kaitlin J. Mayne, Alistair J. Roddick, SMART-C collaboration, Brendon L. Neuen, Sibylle J. Hauske, Stefan D. Anker, Martina Brueckmann, Javed Butler, David Z. I. Cherney, Jennifer B. Green, Chih-Chin Liu, Finnian R. McCausland, Darren K. McGuire, John J. V. McMurray, Milton Packer, Vlado Perkovic, Marc S. Sabatine, Scott D. Solomon, Muthiah Vaduganathan, Christoph Wanner, Stephen D. Wiviott, Faiez Zannad, Hiddo J. L. Heerspink, Sarah Y. A. Ng, Doreen Zhu, Parminder Judge, David Preiss, Martin J. Landray, Colin Baigent, Jonathan R. Emberson, William G. Herrington, Smart-C Steering Committee, Deepak L. Bhatt, David Z. I. Cherney, Bruce Neal, Brendon L. Neuen, Vlado Perkovic, Richard Haynes, William G. Herrington, Hiddo J. L. Heerspink, Silvio E. Inzucchi, Kenneth W. Mahaffey, Darren K. McGuire, John J. V. McMurray, Milton Packer, Marc S. Sabatine, Scott D. Solomon, Muthiah Vadaganathan, Christoph Wanner, Stephen D. Wiviott, David C. Wheeler, Faiez Zannad

**Affiliations:** 1 Renal Studies Group, Medical Research Council Population Health Research Unit at the University of Oxford, Clinical Trial Service Unit and Epidemiological Studies Unit, Nuffield Department of Population Health (NDPH), University of Oxford, Oxford, UK; 2 SGLT- inhibitor Meta-Analysis Cardio-Renal Trialists’ Consortium (SMART-C); 3 Oxford Kidney Unit, Oxford University Hospitals NHS Foundation Trust, Oxford, UK; 4 George Institute for Global Health, Newtown, Australia; University of New South Wales, Sydney, New South Wales, Australia; 5 Vth Department of Medicine, University Medical Center Mannheim, University of Heidelberg, Heidelberg, Germany; 6 Boehringer Ingelheim Pharma GmbH & Co. KG, Ingelheim, Germany; 7 Department of Cardiology (CVK), and Berlin Institute of Health Center for Regenerative Therapies, German Center for Cardiovascular Research Partner Site Berlin, Charité Universitätsmedizin, Berlin, Germany; 8 Faculty of Medicine Mannheim, University of Heidelberg, Mannheim, Germany; 9 Baylor Scott and White Research Institute, TX and University of Mississippi, Jackson, MS, USA; 10 Department of Medicine, University of Toronto, Toronto, Ontario, Canada; 11 Division of Endocrinology, Department of Medicine and Duke Clinical Research Institute, Duke University Medical Center, Durham, NC, USA; 12 Merck & Co., Inc., Rahway, New Jersey, USA; 13 Renal Division (F.R.M.), Brigham and Women’s Hospital, Boston, MA, USA; 14 University of Texas Southwestern Medical Center, Parkland Health and Hospital System, Dallas, TX, USA; 15 British Heart Foundation Cardiovascular Research Centre, University of Glasgow, Glasgow, UK; 16 Baylor Heart and Vascular Institute, Baylor University Medical Center, Dallas, TX, USA; 17 TIMI Study Group; 18 Division of Cardiovascular Medicine, Brigham and Women’s Hospital, Harvard Medical School, Boston, MA, USA; 19 Würzburg University Clinic, Würzburg, Germany; 20 Université de Lorraine, Inserm, Center d’Investigations Cliniques, - Plurithématique 14-33, and Inserm U1116, CHRU, F-CRIN INI-CRCT (Cardiovascular and Renal Clinical Trialists), Nancy, France; 21 Department of Clinical Pharmacy and Pharmacology, University of Groningen, Groningen, the Netherlands; 22 Yale School of Medicine, New Haven, CT, USA; 23 Stanford University School of Medicine, Stanford, CA 94305, USA; 24 Department of Renal Medicine, University College London, London, UK

**Keywords:** sodium glucose co-transporter-2 inhibitors, CKD, AKI, randomised trials

## Abstract

**Background:**

Large trials have shown sodium glucose co-transporter-2 (SGLT2) inhibitors reduce risk of kidney and cardiovascular outcomes in patients with heart failure and chronic kidney disease (CKD), but were not powered to assess outcomes in patients with and without diabetes separately.

**Methods:**

We did a meta-analysis of large placebo-controlled SGLT2 inhibitor trials (PROSPERO:CRD42022351618). The main outcomes were kidney disease progression (standardised to a definition of a sustained ≥50% decline in estimated glomerular filtration rate (eGFR), end-stage kidney disease, or death from kidney failure), acute kidney injury (AKI), mortality and the composite of cardiovascular death or hospitalisation for heart failure.

**Findings:**

13 trials involving a total of 90,413 participants were included (15,605 [17%] without diabetes; trial average baseline eGFR range: 37-85 ml/min/1·73m^2^). Compared with placebo, allocation to an SGLT2 inhibitor reduced the risk of kidney disease progression by 37% (relative risk [RR] 0·63, 95% confidence interval 0·58-0·69) with similar RRs in patients with and without diabetes (heterogeneity p=0·31). In the 4 CKD trials, RRs were similar irrespective of primary kidney diagnoses (heterogeneity p=0·67). SGLT2 inhibitors reduced the risk of AKI by 23% (0·77, 0·70-0·84) and the risk of cardiovascular death or hospitalisation for heart failure by 23% (0·77, 0·74-0·81), again with similar effects in those with and without diabetes (heterogeneity p values=0·12 and 0·67, respectively. Allocation to an SGLT2 inhibitor did not significantly reduce the risk of non-cardiovascular death (0·94, 0·88-1·02), with similar RRs in patients with or without diabetes. For all outcomes, results were also broadly similar irrespective of trial-average baseline eGFR (all trend tests p>0·05). In the trial populations studied to date, the absolute benefits of SGLT2-inhibition outweigh any serious hazards.

**Interpretation:**

The totality of the randomised data supports the use of SGLT2 inhibitors to modify risk of kidney disease progression and AKI, not only in patients with type 2 diabetes, but also in patients with CKD or heart failure irrespective of diabetes status, primary kidney disease or kidney function.

**Funding:**

MRC-UK&KRUK.

## Introduction

Large placebo-controlled trials have demonstrated that sodium glucose co-transporter-2 (SGLT2) inhibitors reduce the risk of cardiovascular disease, and particularly hospitalisation for heart failure, in patients with type 2 diabetes at high risk of atherosclerotic cardiovascular disease (ASCVD), heart failure, or chronic kidney disease (CKD). There is good evidence to support SGLT2 inhibitors as a foundational therapy to prevent cardiovascular death or hospitalisation for heart failure in patients with heart failure irrespective of history of prior diabetes or ejection fraction. (1–5) Large trials have also shown that SGLT2 inhibitors reduce the risk of kidney disease progression in patients with type 2 diabetes and proteinuric CKD, (1, 6–8) but there were relatively few patients with CKD without diabetes in the three previously reported CKD trials. (1) CREDENCE and SCORED exclusively studied patients with CKD with type 2 diabetes, (7, 9) and the DAPA-CKD trial in patients with proteinuric CKD reported just 109 kidney disease progression outcomes in patients without diabetes. (1, 8, 10) Although evidence on the effect of SGLT2 inhibitors on kidney disease progression in patients without diabetes is also available from the heart failure trials - where decreased kidney function was common - previous meta-analysis had limited power as there were only 98 kidney disease progression outcomes in participants without diabetes in such trials. (1, 11)

Two recent placebo-controlled SGLT2 inhibitor trials provide important new information on the effects of kidney disease progression and other outcomes in patients without diabetes. DELIVER randomised 6263 patients with stable heart failure and an ejection fraction >40%, including 3457 (55%) of patients without diabetes (mean estimated glomerular filtration rate [eGFR] 61 mL/min/1·73m^2^), (4) and EMPA-KIDNEY randomised 6609 patients with CKD at risk of progression (mean eGFR 37 mL/min/1·73m^2^), including 3569 (54%) without diabetes. (12) Although there is geographic variation, globally the majority of people with CKD do not have diabetes. (13, 14) There is therefore a need to incorporate these data and perform an updated meta-analysis to summarise definitively the relative and absolute effects of SGLT2 inhibitors on kidney disease progression and other outcomes according to whether or not trial participants had diabetes.

Another limitation of previous meta-analyses has been the inability to standardise between-trial differences in thresholds of eGFR decline used to define categorical kidney disease progression composite outcomes (Webtable 1). (1, 6) We therefore aimed to perform a collaborative meta-analysis assessing the effects of SGLT2 inhibitors on kidney disease progression according to a standardised outcome definition, as well as effects on acute kidney injury (AKI), mortality, heart failure and key safety outcomes by diabetes status. Secondarily, we aimed to assess whether the relative effects of SGLT2 inhibitors on outcomes are modified by mean baseline kidney function (at a trial level) or by primary kidney diagnosis.

## Methods

### Literature search and data extraction

Our outline protocol was registered in the International Prospective Register of Systematic Reviews (PROSPERO) on 5^th^ August 2022 (CRD42022351618). The Preferred Reporting Items for Systematic Reviews and Meta-Analysis (PRISMA) statement was followed. A systematic search of MEDLINE and Embase databases via OVID was performed to cover the period of inception to 5^th^ September 2022. Trials were eligible if they were double-blind and placebo-controlled, performed in adults, and large (defined as ≥500 participants in each arm, thereby minimising any potential for publication bias to distort findings) and at least 6 months in duration. Titles and abstracts were initially screened, with subsequent screening of full texts and risk of bias assessments (using Version 2 of the Cochrane Risk-of-Bias tool (15)) completed independently by two authors (see Webmethods). For each included trial, data were extracted from the principal (3, 4, 7–9, 16–23) and relevant subsidiary peer-reviewed publications (10, 11, 24–40).

The main pre-specified efficacy outcome was a composite kidney disease progression outcome defined as a sustained ≥50% eGFR decline from randomisation, end-stage kidney disease (ESKD, i.e. start of maintenance dialysis or receipt of a kidney transplant), a sustained low eGFR (usually <15 mL/min/1·73m^2^) or death from kidney failure (Webtable 1 provides details). For eight trials this kidney disease progression outcome was unavailable publicly, so individual trial investigators provided a re-analysis of eGFR data to derive this meta-analysis’ pre-selected composite kidney disease progression outcome as well as any other unavailable outcomes of interest (3, 4, 7, 8, 12, 17, 21, 41) (excluding the short duration SOLOIST-WHF trial (18)). Previously reported results mean we now consider AKI an efficacy outcome (rather than a safety outcome). AKI was defined by its specific MedDRA Preferred Term, wherever possible. Other efficacy outcomes were the composite of hospitalisation for heart failure or cardiovascular death (excluding urgent heart failure visits to enable standardisation across trials), cardiovascular mortality (based on individual trial definitions), non-cardiovascular mortality, and all-cause mortality. Safety outcomes were focused on key medical complications that previous meta-analyses have indicated are potentially caused by SGLT2 inhibition: ketoacidosis and lower limb amputation (1) with information on lower limb amputation particularly sought because the CANVAS trial reported a significant excess among participants allocated SGLT2 inhibition. (20) Additional information on urinary tract infections (all and restricted to the subset which are serious), mycotic genital infections, severe hypoglycaemia and bone fractures are included for completeness (Webtable 2 provides details of derivation of each outcome by trial).

For the CKD trials, subgroups by investigator-reported primary kidney diagnosis were grouped as pre-specified in DAPA-CKD and EMPA-KIDNEY into: diabetic kidney disease/nephropathy; ischaemic and hypertensive kidney disease; glomerular disease (also known as glomerulonephritis); and other/unknown combined. (10, 12) CREDENCE excluded suspected non-diabetic kidney disease, and so all participants were considered to have diabetic kidney disease. (7) Based on previous DAPA-CKD publications, (28, 29) exploratory analyses were also conducted by subtype of glomerular disease: IgA nephropathy versus focal segmental glomerulosclerosis versus other glomerulonephritides.

### Statistical analysis

Analyses were performed separately in patients with and without diabetes at baseline (except for analyses by primary kidney diagnosis). Wherever possible, diabetes-specific (or other primary kidney diagnosis-specific) effects of treatment were obtained from Cox models reported in trial publications. Where unavailable (see Webtable 2), log RRs and the associated standard errors (SEs) were estimated from the numbers of events and participants in each arm. Inverse-variance-weighted averages of log hazard ratios/RRs were then used to estimate the treatment effects in each patient group and overall. (42, 43) This information-weighted-average approach has the desirable property that, at the point of randomisation, every participant has the same opportunity to contribute the same amount of statistical information to the meta-analysis as every other participant, without making any assumptions about the nature of any true heterogeneity in results between the trials.

Standard chi-square tests for heterogeneity were used to assess whether treatment effects differed between those with and without diabetes at recruitment, by trial population (based on primary eligibility [Table 1]), and by primary kidney diagnosis. In figures, trials were ordered by their mean baseline eGFR levels and effect modification by kidney function was assessed by a standard test for trend in the set of ordered results. For trials reporting median eGFR and its interquartile range, mean and standard deviation values were estimated. (44) A sensitivity analysis reordering trials by median baseline level of albuminuria was conducted.

Absolute benefits and harms of SGLT2 inhibitors versus placebo per 1000 patient-years of treatment were estimated by diabetes status for each patient group. Absolute effects were estimated by applying the diabetes status-specific RRs, or their 95% confidence limits, to the corresponding mean event rates in the placebo arms (first event only). As in our previous report, (1) data from SOLOIST-WHF were excluded from these analyses due to the extremely high absolute risks in this trial in patients with a recent hospitalisation for heart failure. (18) All analyses were performed in SAS version 9.4 (SAS Institute, Cary NY, USA) and R v3.6.2.

### Role of funding source

The funders had no role in meta-analysis design, analysis, interpretation, writing of the report, or the decision to submit for publication. The senior author accepts full responsibility for the content of the paper.

## Results

### Eligible trial characteristics

Literature searches identified 15 large trials (Webfigure 1). Two trials, one of 1402 participants with type 1 diabetes (inTandem3) and one of 1250 people hospitalised with Coronavirus-19 (DARE-19) were excluded from meta-analyses as follow-up was too short. (1, 23, 45) The remaining 13 trials involved a total of 90,413 randomised patients. All were judged to be at low risk of bias (Webtable 3).

Four trials involving 42,568 patients included people with type 2 diabetes and high-ASCVD risk, five trials involving 21,947 patients included people with heart failure (11,305 with and 10,642 without diabetes), and four trials involving 25,898 patients included people with CKD (20,931 with and 4967 without diabetes) (Table 1/Webtable 4). Average eGFR ranged from 74-85 mL/min/1·73m^2^ in the type 2 diabetes high-ASCVD risk trials, from 50-66 mL/min/1·73m^2^ in the heart failure trials, and from 37-56 mL/min/1·73m^2^ in the CKD trials. Median follow-up was longest for the type 2 diabetes high-ASCVD risk trials (range: 2.4-4·2 years), intermediate for the CKD trials (range: 1·3-2·6 years) and shortest for the heart failure trials (range 0·8-2·2 years).

### Effects on kidney disease outcomes

**Figure 1:**
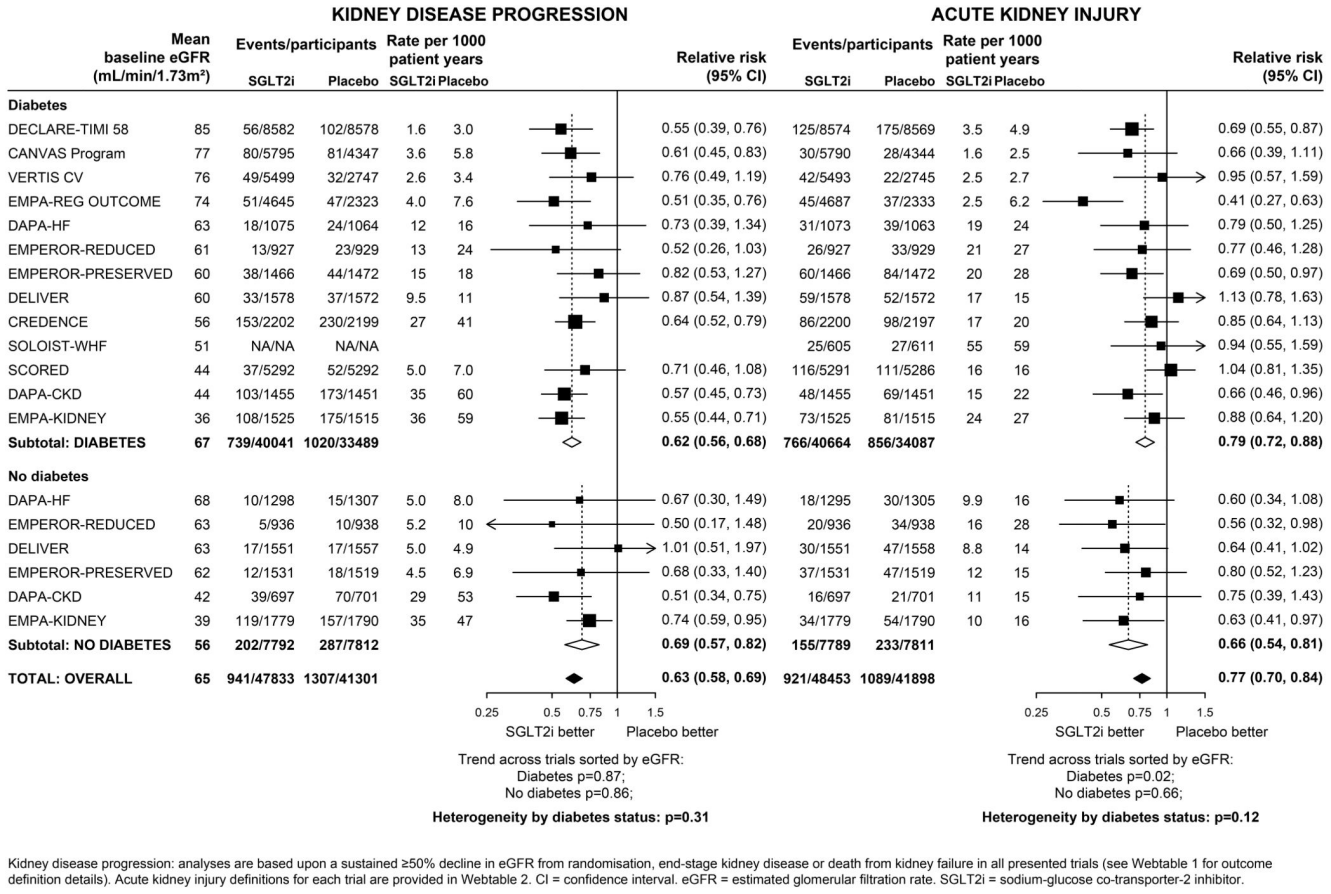
Effect of SGLT2 inhibitors on KIDNEY DISEASE outcomes, by diabetes status

Compared with placebo, allocation to an SGLT2 inhibitor reduced the risk of kidney disease progression by 37% overall (RR 0·63, 95%CI 0·58-0·69; Figure 1). The RR for the kidney failure subcomponent of this outcome overall was 0.67 (0·59-0·77, Webfigure 2). For kidney disease progression, there were similar relative risk reductions in patients with diabetes (0·62, 0·56-0·68) and patients without diabetes (0·69, 0·57-0·82) (heterogeneity p=0·31). There was no evidence that the relative risk reduction varied depending on average baseline eGFR, either in those with diabetes (trend p=0·87) or those without diabetes (trend p=0·86; Figure 1). Nor was there a significant trend in a sensitivity analysis in which trials were reordered by trial median baseline urine albumin-to-creatinine ratio (trend p=1·00 and·0·47 respectively, Webfigure 3).

Suitable data on reported AKI were available from all included trials (Webtable 2). Compared with placebo, allocation to an SGLT2 inhibitor reduced the risk of AKI by 23% overall (0·77, 0·70-0·84), again with similar reductions observed in patients with diabetes (0·79, 0·72-0·88) and patients without diabetes (0·66, 0·54-0·81) (heterogeneity p=0·12). There was no strong evidence for differences in the relative effects by average baseline eGFR (trend p=0·02 in patients with diabetes and p=0·66 for patients without diabetes; Figure 1).

**Figure 2:**
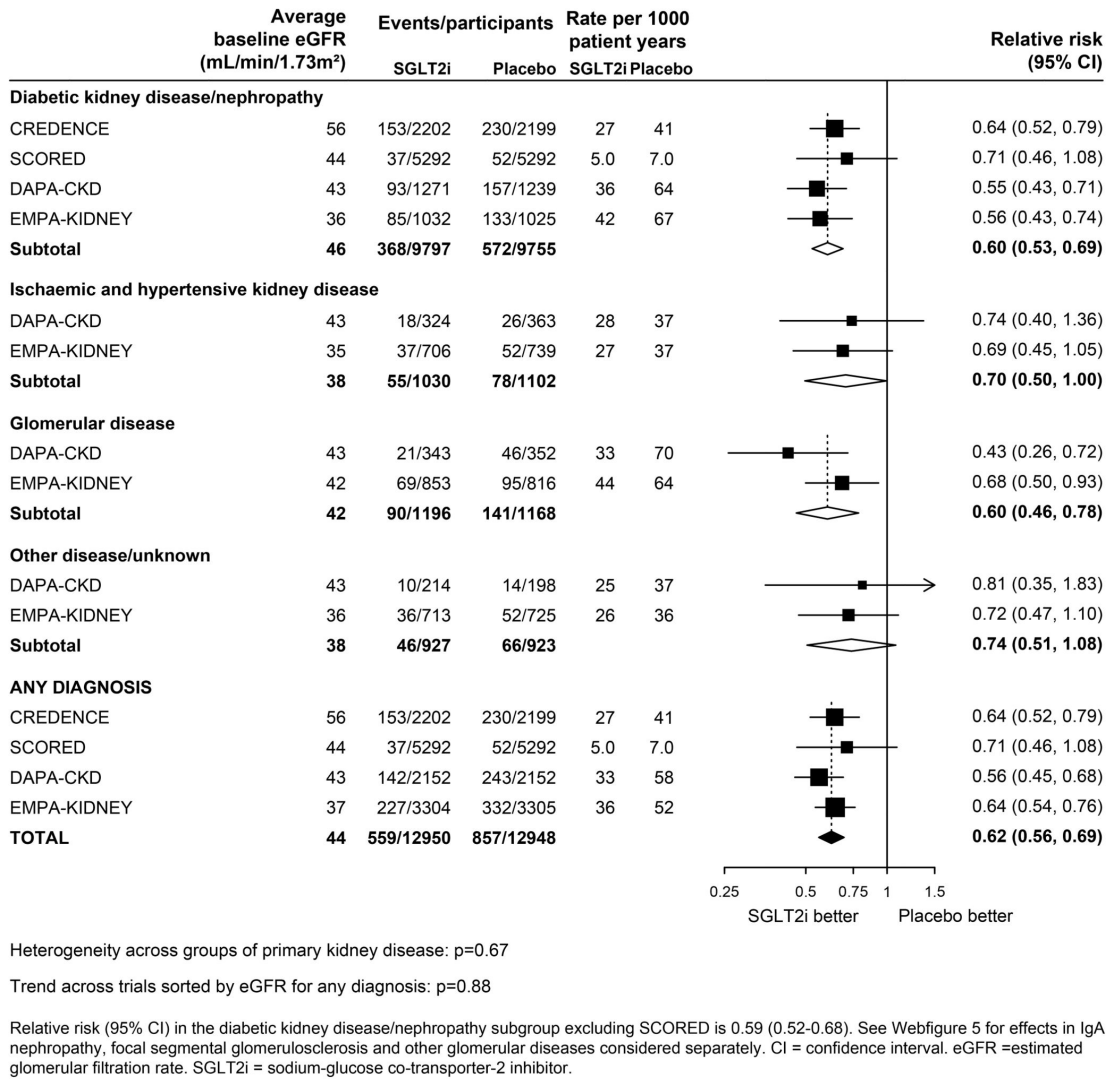
Effect of SGLT2 inhibitors on KIDNEY DISEASE PROGRESSION, by presumed primary kidney disease (CKD trials only)

In the CKD trials, the RRs for kidney disease progression were similar when analyses were split by primary kidney diagnosis (heterogeneity p=0·67; Figure 2). In the four trials that included patients with diabetic kidney disease, SGLT2 inhibitors reduced the risk of kidney disease progression by 40% (0·60, 0·53-0·69). Data from patients with non-diabetic causes of CKD were available from DAPA-CKD and EMPA-KIDNEY. SGLT2 inhibitors reduced the risk of kidney disease progression by 30% (0·70, 0·50-1·00) in patients with ischaemic and/or hypertensive kidney disease, by 40% (0·60, 0·46-0·78) in patients with glomerular diseases, and by 26% (0·74, 0·51-1·08) in patients with other kidney diseases/unknown causes. When glomerular diseases were further split into disease subcategories, there was no evidence of heterogeneity between patients with IgA nephropathy, focal segmental glomerular sclerosis or other glomerulonephritis (heterogeneity p=0·30; Webfigure 5).

### Effects on heart failure and mortality outcomes

**Figure 3:**
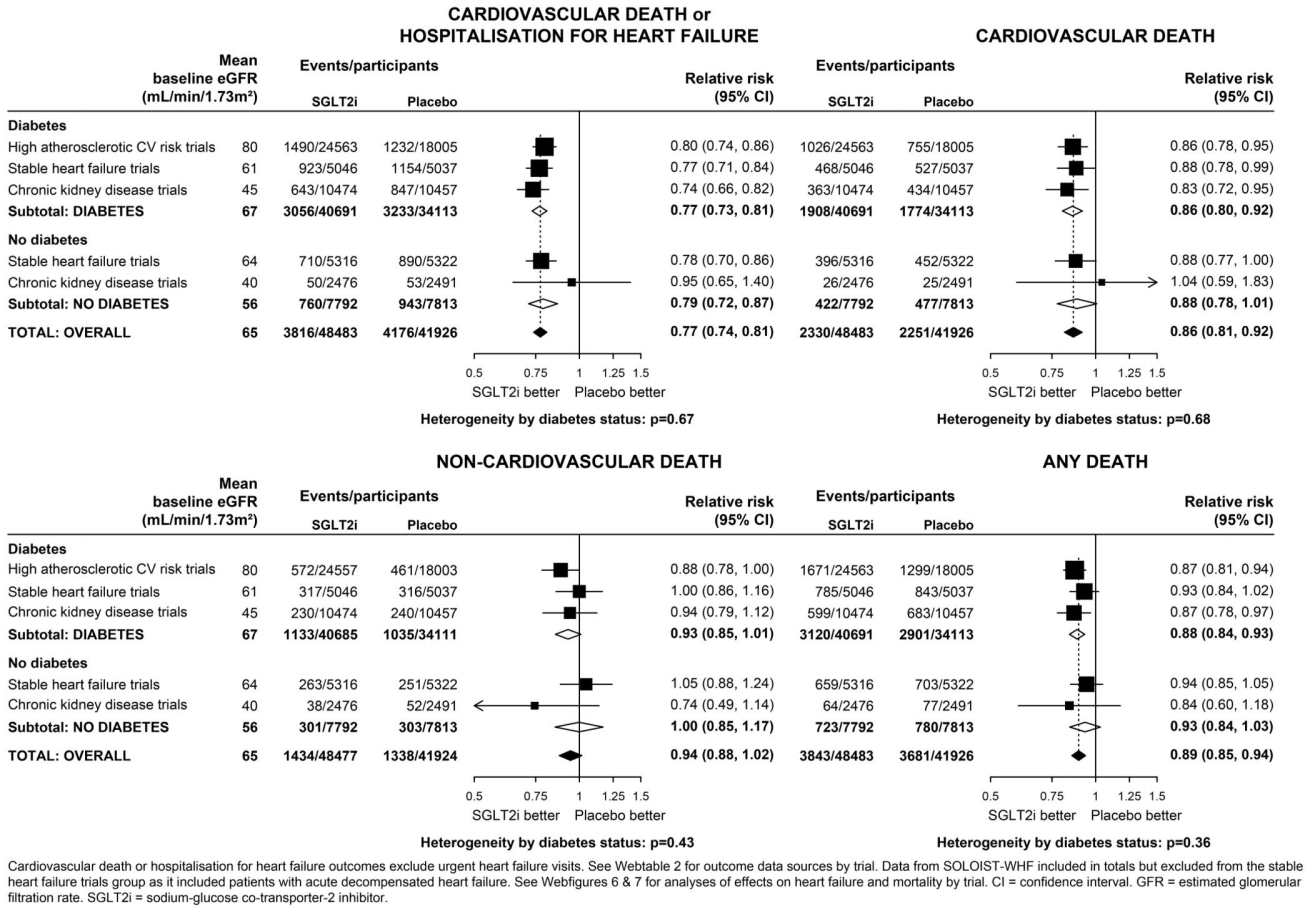
Effect of SGLT2 inhibitors on HEART FAILURE and MORTALITY outcomes, by diabetes status

Overall, compared with placebo, allocation to an SGLT2 inhibitor reduced the risk of the composite of cardiovascular death or hospitalisation for heart failure by 23% (RR 0·77, 0·74-0·81; Figure 3). The RRs were similar irrespective of a history of diabetes (0·77, 0·73-0·81 in patients with diabetes and 0·79, 0·72-0·87 in those without diabetes; heterogeneity p=0·67; Figure 3 and Webfigure 6). Allocation to an SGLT2 inhibitor reduced the risk of cardiovascular death by 14% (0·86, 0·81-0·92), again with similar effects observed in those with diabetes (0·86, 0·80-0·92) and those without diabetes (0·88, 0·78-1·01; heterogeneity p=0·68). Allocation to an SGLT2 inhibitor did not significantly reduce the risk of non-cardiovascular death (0·94, 0·88-1·02), with similar RRs in patients with or without diabetes. There was no evidence that the effects on heart failure or mortality outcomes differed when trial results were ordered by average baseline eGFR (all trend p>0·05; Webfigures 6&7).

### Effects on ketoacidosis, lower limb amputation and other safety outcomes

In patients with diabetes, the absolute risk of ketoacidosis was low (~0.2 per 1000 patient years in placebo arms). The RR for ketoacidosis in patients with diabetes, compared with placebo, allocated to an SGLT2 inhibitor was 2·12 (1·49-3·04) and there was no evidence that this differed when trial results were ordered by average baseline eGFR (trend p=0·69; Webfigure 8). There was only one event of ketoacidosis among patients without diabetes during ~30,000 participant years of follow-up.

**Figure 4:**
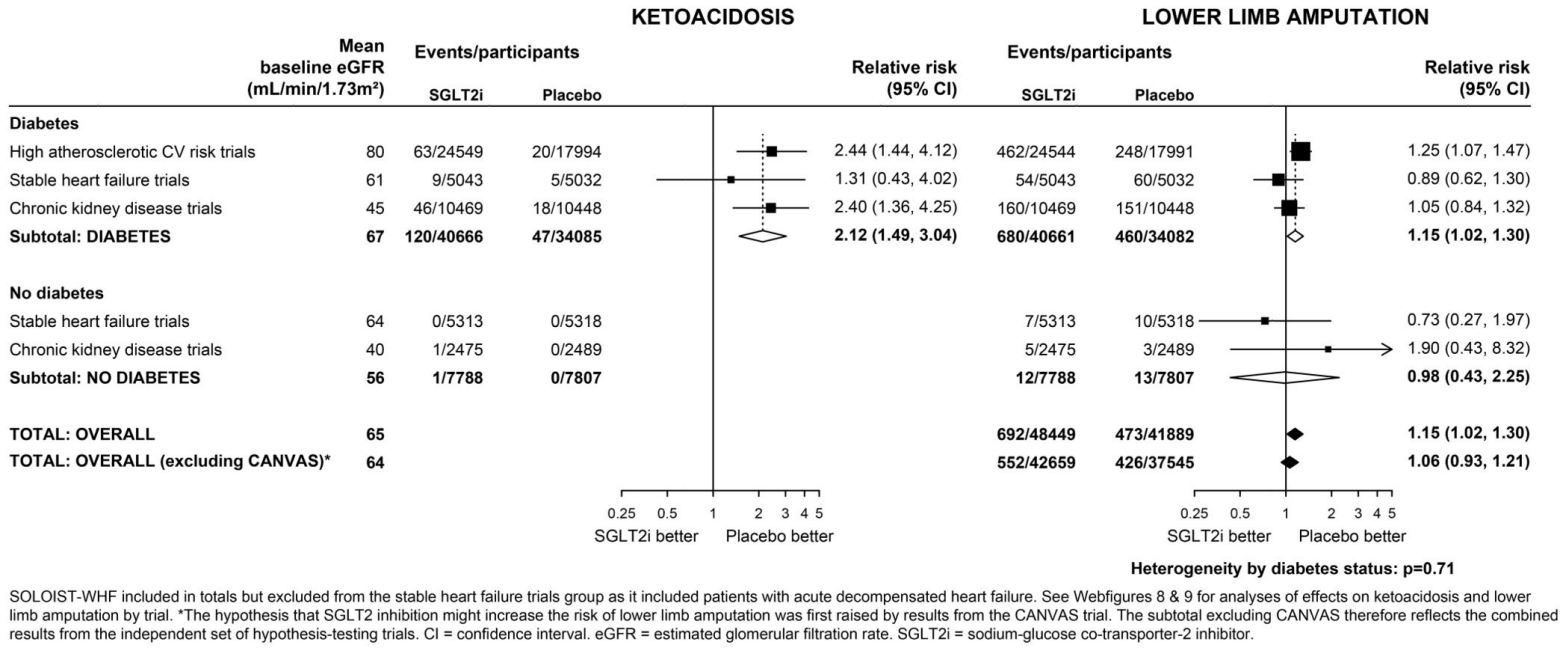
Effect of SGLT2 inhibitors on KETOACIDOSIS and LOWER LIMB AMPUTATION, by diabetes status

In the CANVAS trial, allocation to an SGLT2 inhibitor was associated with a doubling in risk of lower limb amputation (6.3 vs 3.4 per 1000 patients year; Webfigure 9). However in the other 12 trials, allocation to an SGLT2 inhibitor was not significantly associated with lower limb amputation (RR 1·06, 0·93-1·21); Figure 4; heterogeneity p for CANVAS vs other 12 trials <0.001). Across all trials, therefore, allocation to an SGLT2 inhibitor was associated with a 15% increase in the risk of lower limb amputation (RR 1·15, 1·02-1·30). Compared with patients with diabetes, the risk of lower limb amputation was much lower among patients without diabetes. There was no evidence that the RRs for amputations varied depending on average baseline eGFR (trend p>0.05; Webfigure 9). The effects of SGLT2 inhibition on urinary tract infection (1·08, 1·02-1·15), serious urinary tract infection (1·07, 0·90-1·27), mycotic genital infections (3·57, 3·14-4·06), severe hypoglycaemia (0·89, 0·80-0·98) and bone fracture (1·07, 0·99-1·14) are shown in Webfigure 10.

### Estimates of absolute effects of SGLT2 inhibitors

**Figure 5:**
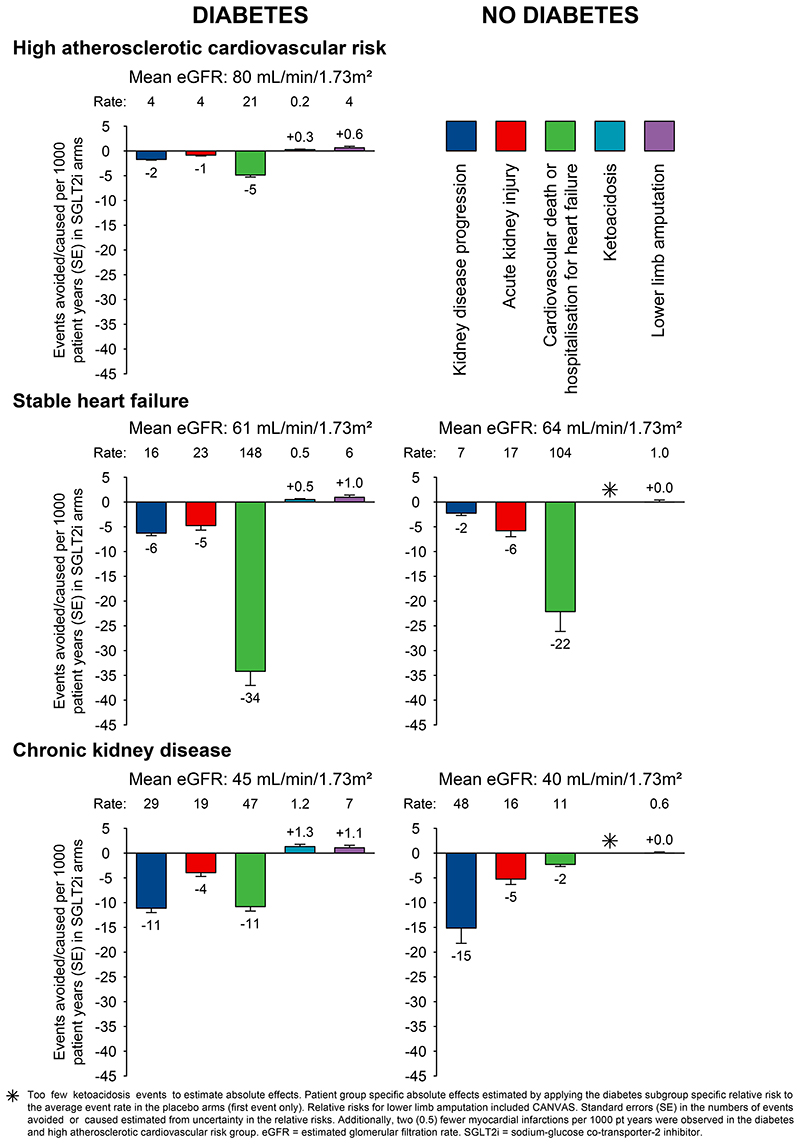
Absolute benefits and harms of SGLT2 inhibitors per 1000 patient years of treatment, by diabetes status and patient group

We estimated absolute rates, benefits and harms of SGLT2 inhibitors by diabetes status and type of trial population (Figure 5). In the studied participants, the absolute risks of kidney disease progression, AKI and cardiovascular death or hospitalisation for heart failure were, generally, slightly lower in patients without diabetes compared to patients with diabetes. Consequently, by population, the absolute benefits were somewhat larger for patients with diabetes. For example, treatment for one year of 1000 patients with CKD and type 2 diabetes with an SGLT2 inhibitor was estimated to result in 11 fewer patients developing kidney disease progression, 4 fewer patients with AKI, and 11 fewer cardiovascular deaths or hospitalisations for heart failure, and cause ~1 episode of ketoacidosis and ~1 lower limb amputation, respectively. The corresponding benefits in patients with CKD without diabetes were 15 fewer patients with kidney disease progression, 5 fewer with AKI, and 2 fewer cardiovascular deaths or hospitalisations for heart failure per 1000 patient-years of treatment, with no excess risk of ketoacidosis or amputation observed.

## Discussion

Large placebo-controlled trials of SGLT2 inhibitors have randomised patients with type 2 diabetes, CKD and heart failure, but no trial was specifically powered to assess kidney or cardiovascular effects in patients without diabetes. Our key objective was to perform a collaborative meta-analysis incorporating all of the available evidence from all large SGLT2 inhibitor trials in CKD, heart failure, and type 2 diabetes at high cardiovascular risk populations to compare definitively their effects on risk of a standardised definition of kidney disease progression, AKI and other key outcomes in patients with and without diabetes. Analyses include information from ~90,000 trial participants, including ~16,000 people without diabetes. Using a definition based on ≥50% sustained decline in eGFR from randomisation, the need to start maintenance dialysis or receive a kidney transplant, sustained low eGFR, or death from kidney disease, our results demonstrate that SGLT2 inhibitors reduce the risk of kidney disease progression by about two-fifths and AKI by about one-quarter, and do so similarly in patients with and without diabetes. Patients with a wide range of kidney function have been studied in the reported trials, and despite attenuation of the effects of SGLT2 inhibitors on glycosuria with lower kidney function, (46) there was no suggestion kidney benefits were attenuated when trials were ordered by average baseline kidney function. SGLT2 inhibitors also appear safe at low levels of kidney function down to at least 20 ml/min/1·73m^2^, with patients without diabetes being at particularly low risk of ketoacidosis or amputation (whether they are receiving an SGLT2 inhibitor or not). In all the trial populations studied to date, the absolute benefits of SGLT2-inhibition considerably outweigh any serious hazards.

The outcome of a sustained ≥50% decline in eGFR from randomisation has been widely used to explore effects on kidney disease progression in subanalyses of the DAPA-CKD trial. (1, 8, 10, 28, 29). This definition appears to be more specific for progression to kidney failure than a sustained ≥40% decline in eGFR for interventions with a negative “acute dip” effect on eGFR, like SGLT2 inhibitors (47–49). The optimal percentage decline in eGFR used to assess kidney disease progression is a trade-off between specificity (increased by larger percentage declines) and outcome event rate (increased by smaller percentage declines). DAPA-CKD suggested the effects of dapagliflozin on kidney disease progression were similar in participants with diabetic kidney disease/nephropathy, glomerular diseases, ischaemic or hypertensive CKD, and CKD of other or unknown cause considered separately. (10, 12) Furthermore, the DAPA-CKD investigators have reported results for 270 patients with IgA nephropathy, the commonest cause of glomerulonephritis worldwide, and reported kidney benefits in this particular subgroup (based on 25 kidney disease progression events). (28) Analyses from EMPA-KIDNEY include a further 817 patients with IgA nephropathy and 80 kidney disease progression outcomes. The current meta-analysis shows that the benefits of SGLT2 inhibitors on kidney disease progression extend to patients irrespective of diabetes status (Figure 1) and in patients with CKD irrespective of their primary cause of kidney disease (Figure 2).

Based on the average risk in different trial populations we estimated that for every 1000 patients with CKD treated for one year with an SGLT2 inhibitor, 11 and 15 first kidney disease progression events would be prevented in patients with and without diabetes, respectively. Such treatment also resulted in an estimated 4-5 fewer AKI events in both patients with and without diabetes. Individual trials have shown that kidney benefits translate into important reductions in the need for dialysis or kidney transplantation (7, 8) (Webfigure 2), and the cardiovascular and kidney benefits appear to be cost saving in diabetic CKD. (50) We found no good evidence that the kidney benefits were modified by the average level of kidney function studied in the trials. Importantly, efficacy and safety data from EMPA-KIDNEY and DAPA-CKD combined include information on nearly 3000 patients with an eGFR between 20-30 mL/min/1·73m^2^. A total of 489 kidney disease progression outcomes accrued in those with an eGFR <30 mL/min/1·73m^2^ in those two trials. (7, 8, 51) Although some clinical practice guidelines have started recommending use of SGLT2 inhibitors in type 2 diabetes at eGFRs down to 20 mL/min/1·73m^2^ (based on grade B levels of evidence), (52, 53) many other recommendations limit initiation to those with eGFR above 25 or 30 mL/min/1·73m^2^. (54–56) As patients with decreased eGFR are at the highest absolute risk of kidney disease progression, (57) our findings should encourage the initiation of SGLT2 inhibitors in patients with CKD down to an eGFR of 20 mL/min/1·73m^2^ with continued use below this level. Furthermore, several hundred participants in the CKD trials had an eGFR below this level both at randomisation (Table 1) or during follow-up, so there is indirect evidence to support nephrologists considering initiation of SGLT2 inhibitors in selected patients with an eGFR below 20 mL/min/1·73m^2^.

This meta-analysis has a number of strengths: it addresses the lack of standardisation of kidney disease progression outcomes in previous meta-analyses and takes into account all of the available large-scale randomised evidence from ~90,000 people recruited into the 13 relevant large placebo-controlled SGLT2 inhibitor clinical trials. The inclusion of new EMPA-KIDNEY and DELIVER data has more than doubled the number of outcomes previously available for kidney disease progression in patients without diabetes. (1) Nevertheless, some limitations remain. First, we found limited numbers of cardiovascular deaths and heart failure hospitalisations in patients with CKD without diabetes: 103 deaths from cardiovascular disease or hospitalisation for heart failure, and 51 cardiovascular deaths. Secondly, adjudication of AKI was not performed in the majority of trials. Thirdly individual participant-level data from all the trials are not yet available, precluding detailed analyses of the rate of change of eGFR (an accepted surrogate of kidney disease progression). (58) Such analyses may provide sufficient power to assess effects of SGLT2 inhibitors in those with slowly progressive CKD where there are more limited data (e.g. patients with CKD with no albuminuria). Fourthly, the efficacy and safety of SGLT2 inhibitors in people with established kidney failure (i.e. requiring dialysis or kidney transplant) remains to be evaluated, (59) and there are insufficient data to assess the effects on kidney and cardiovascular clinical outcomes for patients with other kidney diagnoses excluded from the CKD trials (e.g. polycystic kidney disease) and for patients with type 1 diabetes (see Web Methods for inTandem3 data). (23, 60) Lastly, our absolute effect estimates are specific to the recruited trial populations. RRs are more generalisable, and so, in routine clinical practice, absolute effects of SGLT2 inhibitors could be estimated for an individual by calculating their absolute risk for an event using an established risk score and then applying the RRs for the relevant outcome from the present meta-analysis.

In conclusion, our meta-analysis of all the large placebo-controlled SGLT2 inhibitor trials has shown that SGLT2 inhibitors safely reduce risk of kidney disease progression, AKI, cardiovascular death and hospitalisation for heart failure in patients with CKD or heart failure, irrespective of diabetes status. On a relative scale, these benefits are similar in patients with and without diabetes and appeared to be evident across the wide range of kidney function studied. Combining the two large trials in CKD populations to recruit patients with non-diabetic causes of kidney disease (EMPA-KIDNEY and DAPA-CKD), we also found relative benefits on kidney disease progression appeared similar across the range of primary kidney diagnoses studied. Large trials support a central role for SGLT2 inhibitors as disease-modifying therapy for treatment of CKD, irrespective of diabetes status, primary kidney diagnosis, or level of kidney function.

## Supplementary Material

Appendix

## Figures and Tables

**Table 1:** Summary of included trials

Patient group Trial acronym(drug & daily dose)	Size	Median follow-up, years	Proportion with diabetes n (%)	Proportion with heart failure n (%)	Mean (SD) eGFR, mL/min/1.73m^2^	Median (IQR) urinary ACR, mg/g	Key eligibility criteria
**Type 2 diabetes at high ASCVD risk**							
DECLARE-TIMI 58 (dapagliflozin 10mg)	17160	4.2	17160 (100)	1724 (10)	85 (16)	13.1 (6.0-43.6)	Type 2 diabetes Age 40y + history of coronary, cerebral or peripheral vascular disease OR age ≥55y in men/≥60y in women with at least 1 CV risk factor Creatinine clearance ≥60 mL/min
CANVAS Program (canagliflozin 100-300mg)	10142	2.4	10142 (100)	1461 (14)	77 (21)	12.3 (6.7-42.1)	Type 2 diabetes History of coronary, cerebral or peripheral vascular disease OR age >50y with at least 2 CV risk factors eGFR ≥30
VERTIS CV (ertugliflozin 5 or 15 mg)	8246	3.0	8246 (100)	1958 (24)	76 (21)	19.0 (6.0-68.0)	Type 2 diabetes History of coronary, cerebral or peripheral vascular disease eGFR ≥30
EMPA-REG OUTCOME (empagliflozin 10mg or 25mg)	7020	3.1	7020 (100)	706 (10)	74 (21)	17.7 (7.1-72.5)	Type 2 diabetes History of coronary, cerebral or peripheral vascular disease eGFR ≥30
**Heart failure**							
DAPA-HF (dapagliflozin 10mg)	4744	1.5	2139 (45)^*^	4744 (100)	66 (19)	NA	Symptomatic chronic HF (class II-IV) with LVEF ≤40%(i.e. reduced ejection fraction) o NT-proBNP ≥600 pg/mL o eGFR ≥30 o Appropriate doses of medical therapy & use of medical devices
EMPEROR-REDUCED (empagliflozin 10mg)	3730	1.3	1856 (50)	3730 (100)	62 (22)	22.1 (8.0-81.3)	Class II-IV chronic HF with LVEF ≤40%(i.e. reduced ejection fraction) NT-proBNP above a certain threshold (stratified by LVEF) Appropriate doses of medical therapy and use of medical devices
EMPEROR-PRESERVED (empagliflozin 10mg)	5988	2.2	2938 (49)	5988 (100)	61 (20)	21.0 (8.0-71.6)	Symptomatic chronic HF (class II-IV) with LVEF >40% Echocardiographic evidence of structural heart disease or hospitalisation for heart failure in the last year NT-proBNP >300 pg/mL (or >900 pg/mL if in AF) eGFR ≥20 No recent coronary event
DELIVER (dapagliflozin 10mg)	6263	2.3	3150 (50)^†^	6263 (100)	61 (19)	NA	Symptomatic HF (class II-IV) with LVEF >40% (ambulatory or hospitalised) Echocardiographic evidence of structural heart disease NT-proBNP ?300 pg/mL (or ≥600 pg/mL if in AF)
SOLOIST-WHF (sotagliflozin 200-400mg)	1222	0.8	1222 (100)	1222 (100)	51 (17)^$^	NA	Hospitalised for HF requiring intravenous therapy (i.e. a HF population with a wide range of LVEFs) Type 2 diabetes eGFR ≥30 No recent coronary event
**Chronic kidney disease**							
CREDENCE (canagliflozin 100mg)	4401	2.6	4401 (100)	652 (15)	56 (18)	927 (463-1833)	Type 2 diabetes eGFR 30-90 uACR 300-5000 mg/g Stable maximally tolerated RAS blockade Excluded suspected non-diabetic kidney disease
SCORED (sotagliflozin 200-400mg)	10584	1.3	10584 (100)	3283 (31)	44 (11)^$^	74 (17-481)	Type 2 diabetes eGFR 25-60 At least 1 CV risk factor
DAPA-CKD (dapagliflozin 10mg)	4304	2.4	2906 (68)	468 (11)	43 (12)	949 (477-1885)	eGFR 25-75 uACR 200-5000 mg/g Stable maximally tolerated RAS blockade, unless documented intolerance Excluded polycystic kidney disease, lupus nephritis, or anti-neutrophil cytoplasmic antibody-associated vasculitis.
EMPA-KIDNEY(empagliflozin 10mg)	6609	2.0	3040 (46) ^†^	658 (10)	37.3 (14)	329 (49-1069)	eGFR 20-45 or eGFR 45-90 with uACR ≥200 mg/g at screening^‡^ Clinically appropriate RAS blockade, unless not indicated or not tolerated Excluded polycystic kidney disease

‡ 254 participants with an eGFR<20mL/min/1·73m^2^ at randomisation and 68 with type 1 diabetes.

* Includes patients with HbA1c ≥6.5% at enrolment.

† Includes patients with HbA1c ≥6.5% at baseline or history and/or prevalent use of a glucose-lowering agent.

$ The mean and SD were estimated from reported median and IQR.

AF = atrial fibrillation; ASCVD = atherosclerotic cardiovascular disease; CV = cardiovascular; eGFR = estimated glomerular filtration rate (mL/min/1.73m^2^); HF = heart failure; LVEF = left ventricular ejection fraction; NT-proBNP = N-terminal prohormone brain natriuretic peptide; RAS = renin angiotensin system; uACR = urinary albumin:creatinine ratio.

## Data Availability

Analysed data were extracted from published sources or provided by individual co-authors (see Contributions section). For the purpose of open access, the author(s) has applied a Creative Commons Attribution (CC BY) licence to any Author Accepted Manuscript version arising.

## Research in Context

### Evidence before this study

Our previous meta-analysis reported in 2021 included 11 large placebo-controlled trials conducted in a range of different at-risk populations, and demonstrated that overall, sodium glucose co-transporter-2 (SGLT2) inhibitors reduced risk of kidney disease progression and the composite of cardiovascular death or hospitalisation for heart failure, both by about one-quarter. Relative risks were remarkably consistent across these different types of patient groups. However, data were much more limited in patients without diabetes who were eligible for inclusion in only one of the reported trials in patients with chronic kidney disease (CKD), and three trials in patients with heart failure. Estimates of the effects of SGLT2 inhibitors on kidney disease progression in patients without diabetes were based on only ~100 events from the CKD trial and ~100 events from the heart failure trials. This limits the quality of evidence on which to make clinical practice recommendations. The impact of diabetes on the effects of SGLT2 inhibitors on AKI, mortality and safety outcomes was also not explored.

### Added value of this study

The majority of people with CKD do not have diabetes, so more information about SGLT2 inhibitors in this patient group has particular public health importance. Since 2021, two placebo-controlled SGLT2 inhibitor trials (EMPA-KIDNEY & DELIVER) have studied a large number of people without diabetes. EMPA-KIDNEY recruited 6609 patients with CKD including 3569 patients without diabetes, while DELIVER recruited 6263 patients with heart failure with mildly reduced or preserved (>40%) ejection fraction including 3457 patients without diabetes. Incorporating data from these trials and standardising outcome definitions, this updated meta-analysis definitively shows that in patients with CKD or heart failure (where CKD was common), SGLT2 inhibitors safely reduced the relative risks of kidney disease progression by about 40% and AKI by nearly a quarter, irrespective of diabetes status. Benefits on kidney disease progression also appeared similar across the full range of studied kidney function, and appeared unmodified by primary kidney diagnosis.

### Implications of all the available evidence

This meta-analysis provides high-quality evidence to support guideline recommendations for use of SGLT2 inhibitors as a foundational therapy to reduce risks of kidney disease progression and AKI not only in at-risk patients with type 2 diabetes, but also in patients who have CKD or heart failure (irrespective of diabetes status, primary kidney diagnosis, or level of kidney function).
