# Impact of diabetes on the effects of sodium glucose co-transporter-2 inhibitors on kidney outcomes: collaborative meta-analysis of large placebo-controlled trials

**DOI:** 10.1016/S0140-6736(22)02074-8

**Published:** 2022-11-19

**Authors:** The Nuffield Department of Population Health Renal Studies Group, Colin Baigent, JonathanR. Emberson, Richard Haynes, William G. Herrington, Parminder Judge, Martin J. Landray, Kaitlin J. Mayne, Sarah Y.A. Ng, David Preiss, Alistair J. Roddick, Natalie Staplin, Doreen Zhu, SGLT2 inhibitor Meta-Analysis Cardio-Renal Trialists' Consortium, Stefan D. Anker, Deepak L. Bhatt, Martina Brueckmann, Javed Butler, David Z.I. Cherney, Jennifer B. Green, Sibylle J. Hauske, Richard Haynes, Hiddo J.L. Heerspink, William G. Herrington, Silvio E. Inzucchi, Meg J. Jardine, Chih-Chin Liu, Kenneth W. Mahaffey, Finnian R. McCausland, Darren K. McGuire, John J.V. McMurray, Bruce Neal, Brendon L. Neuen, Milton Packer, Vlado Perkovic, Marc S. Sabatine, Scott D. Solomon, Muthiah Vaduganathan, Christoph Wanner, David C. Wheeler, Stephen D. Wiviott, Faiez Zannad

## Abstract

**Background:**

Large trials have shown that sodium glucose co-transporter-2 (SGLT2) inhibitors reduce the risk of adverse kidney and cardiovascular outcomes in patients with heart failure or chronic kidney disease, or with type 2 diabetes and high risk of atherosclerotic cardiovascular disease. None of the trials recruiting patients with and without diabetes were designed to assess outcomes separately in patients without diabetes.

**Methods:**

We did a systematic review and meta-analysis of SGLT2 inhibitor trials. We searched the MEDLINE and Embase databases for trials published from database inception to Sept 5, 2022. SGLT2 inhibitor trials that were double-blind, placebo-controlled, performed in adults (age ≥18 years), large (≥500 participants per group), and at least 6 months in duration were included. Summary-level data used for analysis were extracted from published reports or provided by trial investigators, and inverse-variance-weighted meta-analyses were conducted to estimate treatment effects. The main efficacy outcomes were kidney disease progression (standardised to a definition of a sustained ≥50% decrease in estimated glomerular filtration rate [eGFR] from randomisation, a sustained low eGFR, end-stage kidney disease, or death from kidney failure), acute kidney injury, and a composite of cardiovascular death or hospitalisation for heart failure. Other outcomes were death from cardiovascular and non-cardiovascular disease considered separately, and the main safety outcomes were ketoacidosis and lower limb amputation. This study is registered with PROSPERO, CRD42022351618.

**Findings:**

We identified 13 trials involving 90 413 participants. After exclusion of four participants with uncertain diabetes status, we analysed 90 409 participants (74 804 [82·7%] participants with diabetes [>99% with type 2 diabetes] and 15 605 [17·3%] without diabetes; trial-level mean baseline eGFR range 37–85 mL/min per 1·73 m^2^). Compared with placebo, allocation to an SGLT2 inhibitor reduced the risk of kidney disease progression by 37% (relative risk [RR] 0·63, 95% CI 0·58–0·69) with similar RRs in patients with and without diabetes. In the four chronic kidney disease trials, RRs were similar irrespective of primary kidney diagnosis. SGLT2 inhibitors reduced the risk of acute kidney injury by 23% (0·77, 0·70–0·84) and the risk of cardiovascular death or hospitalisation for heart failure by 23% (0·77, 0·74–0·81), again with similar effects in those with and without diabetes. SGLT2 inhibitors also reduced the risk of cardiovascular death (0·86, 0·81–0·92) but did not significantly reduce the risk of non-cardiovascular death (0·94, 0·88–1·02). For these mortality outcomes, RRs were similar in patients with and without diabetes. For all outcomes, results were broadly similar irrespective of trial mean baseline eGFR. Based on estimates of absolute effects, the absolute benefits of SGLT2 inhibition outweighed any serious hazards of ketoacidosis or amputation.

**Interpretation:**

In addition to the established cardiovascular benefits of SGLT2 inhibitors, the randomised data support their use for modifying risk of kidney disease progression and acute kidney injury, not only in patients with type 2 diabetes at high cardiovascular risk, but also in patients with chronic kidney disease or heart failure irrespective of diabetes status, primary kidney disease, or kidney function.

**Funding:**

UK Medical Research Council and Kidney Research UK.

## Introduction

Large placebo-controlled trials have shown that sodium glucose co-transporter-2 (SGLT2) inhibitors reduce the risk of cardiovascular disease, and particularly hospitalisation for heart failure, in patients with type 2 diabetes at high risk of atherosclerotic cardiovascular disease, heart failure, or chronic kidney disease. There is good evidence to support the use of SGLT2 inhibitors as a foundational therapy to prevent cardiovascular death or hospitalisation for heart failure in patients with heart failure, irrespective of ejection fraction or history of previous diabetes.^1^, ^2^, ^3^, ^4^, ^5^ Large trials have also shown that SGLT2 inhibitors reduce the risk of kidney disease progression in patients with type 2 diabetes and proteinuric chronic kidney disease,^1^, ^6^, ^7^, ^8^ although few patients with chronic kidney disease without diabetes were included in the three previously reported chronic kidney disease trials.^1^ CREDENCE and SCORED exclusively studied patients with chronic kidney disease and type 2 diabetes,^7^, ^9^ and the DAPA-CKD trial in patients with proteinuric chronic kidney disease reported just 109 kidney disease progression outcomes in patients without diabetes.^1^, ^8^, ^10^ Although evidence of the effect of SGLT2 inhibitors on kidney disease progression in patients without diabetes is also available from the reported heart failure trials (in which decreased kidney function was common), a previous meta-analysis had limited power as only 98 kidney disease progression outcomes were reported in participants without diabetes in such trials.^1^, ^11^

**Research in context**

**Evidence before this study**

In our previous systematic review and meta-analysis reported in 2021, we systematically searched MEDLINE and Embase from inception to Aug 28, 2021, for large double-blind placebo-controlled sodium glucose co-transporter-2 (SGLT2) inhibitor trials. We identified 11 large trials with low risk of bias conducted in three at-risk populations (type 2 diabetes and high atherosclerotic cardiovascular risk, heart failure, and chronic kidney disease). Overall, SGLT2 inhibitors reduced the risk of kidney disease progression and the composite of cardiovascular death or hospitalisation for heart failure, both by about a quarter. Relative risks were markedly consistent across the different patient groups. However, data were limited in patients without diabetes who were eligible for inclusion (in one trial in patients with chronic kidney disease and three trials in patients with heart failure). Estimates of the effects of SGLT2 inhibitors on kidney disease progression in patients without diabetes were based on around 100 events from the chronic kidney disease trial and around 100 events from the heart failure trials. This limits the quality of evidence for making clinical practice recommendations. The influence of diabetes on the effects of SGLT2 inhibitors on acute kidney injury, cardiovascular and non-cardiovascular mortality, and safety outcomes was also not explored in the previous meta-analysis.

**Added value of this study**

The majority of people with chronic kidney disease do not have diabetes, and thus more information about SGLT2 inhibitors in this patient group has particular public health importance. Since 2021, two placebo-controlled SGLT2 inhibitor trials (EMPA-KIDNEY and DELIVER) have studied a large number of people without diabetes. EMPA-KIDNEY recruited 6609 patients with chronic kidney disease including 3569 patients without diabetes, and DELIVER recruited 6263 patients with heart failure with mildly reduced or preserved (>40%) ejection fraction including 3109 patients without diabetes. By incorporating data from these trials and standardising outcome definitions, the current updated meta-analysis shows that in the studied patients with chronic kidney disease or heart failure (in whom chronic kidney disease was common), SGLT2 inhibitors safely reduced the risk of kidney disease progression by 37% (relative risk 0·63, 95% CI 0·58–0·69) and of acute kidney injury by 23% (0·77, 0·70–0·84), compared with placebo, with similar reductions in patients with and without diabetes. Apparent benefits on kidney disease progression were also similar across the range of studied kidney function, and appeared unmodified by primary kidney diagnosis.

**Implications of all the available evidence**

This meta-analysis provides high-quality evidence to support guideline recommendations for the use of SGLT2 inhibitors as a foundational therapy to reduce the risk of kidney disease progression and acute kidney injury not only in patients with type 2 diabetes at high cardiovascular risk, but also in patients who have chronic kidney disease or heart failure, irrespective of diabetes status, primary kidney diagnosis, or level of kidney function.

Two recent placebo-controlled SGLT2 inhibitor trials have provided important new information on the effects of SGLT2 inhibitors on kidney disease progression and other outcomes in patients without diabetes. DELIVER randomly assigned 6263 patients with stable heart failure and an ejection fraction of greater than 40%, including 3109 (49·6%) patients without diabetes (mean estimated glomerular filtration rate [eGFR] 61 mL/min per 1·73 m^2^),^4^ and EMPA-KIDNEY randomly assigned 6609 patients with chronic kidney disease at risk of progression (mean eGFR 37 mL/min per 1·73 m^2^), including 3569 (54·0%) without diabetes.^12^, ^13^ Although geographical variation exists, globally the majority of people with chronic kidney disease do not have diabetes.^14^, ^15^ Therefore, these data need to be incorporated and an updated meta-analysis performed to definitively summarise the relative and absolute effects of SGLT2 inhibitors on kidney disease progression and other outcomes according to whether or not trial participants had diabetes.

Another limitation of previous meta-analyses was the inability to standardise between-trial differences in the thresholds of eGFR decrease used to define kidney disease progression within categorical composite outcomes (appendix p 9).^1^, ^6^ We therefore performed a collaborative meta-analysis assessing the effects of SGLT2 inhibitors on kidney disease progression according to a standardised outcome definition, and on acute kidney injury, death, heart failure, and key safety outcomes by diabetes status. Secondarily, we assessed whether the relative effects of SGLT2 inhibitors on outcomes are modified by mean baseline kidney function (at a trial level) or by primary kidney diagnosis.

## Methods

### Search strategy and selection criteria

We followed the Preferred Reporting Items for Systematic Reviews and Meta-Analyses checklist in the conduct and reporting of this study. We did a systematic search of the MEDLINE and Embase databases via Ovid to cover the period from database inception to Sept 5, 2022. Further details and search terms are listed in the appendix (pp 3–7). Trials were eligible if they assessed SGLT2 inhibitors (including combined SGLT1/2 inhibitors) and if they were double-blind and placebo-controlled (excluding crossover trials), performed in adults (age ≥18 years), large (defined as ≥500 participants in each arm, thereby minimising any potential for publication bias to distort findings), at least 6 months in duration, and reported any of the prespecified efficacy or safety outcomes. Titles and abstracts were initially screened for relevance and duplicates by one author (AJR). The EMPA-KIDNEY trial baseline report^12^ was available while the final report^13^ was unpublished at the time of the search. Subsequent screening of full texts and risk of bias assessments (with version 2 of the Cochrane Risk-of-Bias tool^16^) were completed independently by two authors (KJM, AJR) with conflicts resolved by consensus discussion.

### Data analysis

For each included trial, summary data were extracted from the principal and relevant subsidiary peer-reviewed publications, independently and in duplicate by two authors (KJM, AJR) with discrepancies resolved by consensus discussion (appendix p 4). For trials without previously published relevant outcomes, results were provided by trial investigators.

The main focus of efficacy analyses was on kidney disease progression, acute kidney injury, and a composite of cardiovascular death or hospitalisation for heart failure. Kidney disease progression was defined as a sustained eGFR decrease (≥50%) from randomisation, end-stage kidney disease (ie, start of maintenance dialysis or receipt of a kidney transplant), a sustained low eGFR (<15 mL/min per 1·73 m^2^ or <10 mL/min per 1·73 m^2^) or death from kidney failure (appendix p 9). For eight trials this kidney disease progression outcome was unavailable publicly, and thus individual trial investigators provided a re-analysis of eGFR data to derive our composite kidney disease progression outcome and any other unavailable outcomes of interest^3^, ^4^, ^7^, ^8^, ^12^, ^17^, ^18^, ^19^ (data unavailable from the short duration SOLOIST-WHF trial^20^). The kidney failure component of the primary outcome was defined as a composite of maintenance dialysis, kidney transplantation, or sustained low eGFR. On the basis of previously reported results, we considered acute kidney injury an efficacy outcome (rather than a safety outcome). Acute kidney injury was defined by its specific Medical Dictionary for Regulatory Activities Preferred Term, wherever possible (appendix pp 10–11). The composite of hospitalisation for heart failure or cardiovascular death excluded urgent heart failure visits to enable standardisation across trials. Cardiovascular and non-cardiovascular death were also assessed and retained individual trial definitions. All-cause mortality is a less generalisable outcome than cause-specific mortality, but it was included for completeness. Safety outcomes focused on key medical complications that previous meta-analyses have indicated are potentially caused by SGLT2 inhibition: ketoacidosis and lower limb amputation,^1^ with information on lower limb amputation particularly sought because the CANVAS trial reported a significant excess of amputation among participants allocated to SGLT2 inhibition.^21^ Additional information on urinary tract infections (all infections and restricted to serious infections), mycotic genital infections, severe hypoglycaemia, and bone fractures was included for completeness. Details on the derivation of each outcome by trial are provided in the appendix (pp 10–11).

For the trials in chronic kidney disease, we used prespecified subgroups according to investigator-reported primary kidney diagnosis when possible. This applied for DAPA-CKD and EMPA-KIDNEY, with the subgroups: diabetic kidney disease or nephropathy; ischaemic and hypertensive kidney disease; glomerular disease (also known as glomerulonephritis); and other kidney disease or diagnosis or unknown combined.^10^, ^12^, ^13^ CREDENCE excluded suspected non-diabetic kidney disease, and so all participants were considered to have diabetic kidney disease.^7^ A sensitivity analysis excluding SCORED was conducted due to an absence of data on investigator-reported primary kidney diagnosis.^9^ On the basis of previous DAPA-CKD publications,^22^, ^23^ exploratory analyses were also conducted by subtype of glomerular disease: immunoglobulin A (IgA) nephropathy versus focal segmental glomerulosclerosis versus other glomerulonephritides.

Analyses were done separately in patients with and without diabetes at baseline (except for analyses by primary kidney diagnosis). When possible, diabetes-specific (or primary kidney diagnosis-specific) effects of treatment were obtained from Cox models reported in trial publications. When these effects were unavailable (appendix pp 10–11), log relative risk (RR) and the associated SE were estimated from the numbers of events and participants in each arm. Inverse-variance-weighted averages of log hazard ratios or log RRs were then used to estimate the treatment effects, summarised as RR (95% CI), in each patient group and overall.^24^, ^25^ This information-weighted-average approach has the desirable property that, at the point of random assignment, every participant has the same opportunity to contribute the same amount of statistical information to the meta-analysis as every other participant, without making any assumptions about the nature of any true heterogeneity in results between the trials.

Tests of between-study heterogeneity were conducted in our previous meta-analysis published in 2021, which established that effects were generally similar across its included trials (excluding DELIVER and EMPA-KIDNEY).^1^ Standard χ^2^ tests for heterogeneity were used to assess whether treatment effects differed between those with and without diabetes at recruitment, by trial population (based on predefined trial eligibility [table]) and by primary kidney diagnosis. Heterogeneity was also assessed post hoc for the lower limb amputation outcome, comparing CANVAS with the other 12 trials combined. In forest plots, trials were ordered by their mean baseline eGFR values and effect modification by kidney function was assessed by a standard χ^2^ test for trend across the set of ordered results. Heterogeneity and trend p values were interpreted in the context of the multiple exploratory hypotheses being tested and the absence of individual participant-level data. For trials reporting median eGFR and its IQR, mean and SD values were estimated.^43^ A sensitivity analysis reordering trials by median baseline level of albuminuria (urine albumin-to-creatinine ratio) was conducted.

Rates of outcome events were presented per 1000 patient-years. For the outcomes of kidney disease progression, acute kidney injury, cardiovascular death or hospitalisation for heart failure, ketoacidosis, and lower limb amputation, absolute benefits and harms of SGLT2 inhibitors versus placebo per 1000 patient-years were estimated by diabetes status and patient group. Absolute effects were estimated by applying the diabetes status-specific RRs, or their 95% CIs, to the corresponding mean event rates in the placebo arms (first event only). As in our previous report,^1^ data from SOLOIST-WHF were excluded from these analyses due to the extremely high absolute risks in this trial in patients with a recent hospitalisation for heart failure.^20^

All analyses were performed in SAS (version 9.4) and R (version 3.6.2). Our outline protocol was registered in PROSPERO on Aug 5, 2022 (CRD42022351618).

### Role of funding source

The funders of the study had no role in study design, data collection, data analysis, data interpretation, or writing of the report.

## Results

Our literature searches identified 15 large trials (appendix p 14). Two large trials, one with 1402 participants with type 1 diabetes (the inTandem3 trial) and one with 1250 people hospitalised with COVID-19 (the DARE-19 trial) were excluded from meta-analyses as follow-up was less than 6 months (appendix p 8).^1^, ^44^, ^45^ Results from the remaining 13 trials' main reports^3^, ^4^, ^7^, ^8^, ^9^, ^13^, ^17^, ^18^, ^20^, ^21^, ^30^, ^31^, ^34^ (and their relevant subsidiary publications^10^, ^11^, ^22^, ^23^, ^26^, ^27^, ^28^, ^29^, ^32^, ^33^, ^35^, ^36^, ^37^, ^38^, ^39^, ^40^, ^41^, ^42^, ^46^) included a total of 90 413 randomly assigned patients. 32 238 (35·7%) patients were women and trial-level mean age ranged from 61·9 years to 71·8 years (appendix p 12). All 13 trials were judged to be at low risk of bias (appendix p 13).

Four trials included 42 568 patients with type 2 diabetes and high risk of atherosclerotic cardiovascular disease; five trials included 21 947 patients with heart failure (11 305 with diabetes, 10 638 without diabetes, and four with uncertain status); and four trials included 25 898 patients with chronic kidney disease (20 931 with diabetes and 4967 without diabetes; table). Patients with uncertain diabetes status were excluded from all analyses, resulting in 90 409 patients in the final analysis population. More than 99% of participants with diabetes had type 2 diabetes. The range of values for trial-level mean baseline eGFR was 74–85 mL/min per 1·73 m^2^ in the type 2 diabetes and high atherosclerotic cardiovascular disease risk trials, 51–66 mL/min per 1·73 m^2^ in the heart failure trials, and 37–56 mL/min per 1·73 m^2^ in the chronic kidney disease trials. Median follow-up was longest for the type 2 diabetes and high atherosclerotic cardiovascular disease risk trials (2·4–4·2 years), intermediate for the chronic kidney disease trials (1·3–2·6 years), and shortest for the heart failure trials (0·8–2·2 years).

**Table:** Summary of included trials

	**Size, n**	**Median follow-up, years**	**Proportion with diabetes, n (%)**	**Proportion with heart failure, n (%)**	**Mean (SD) eGFR, mL/min per1·73 m^2^**	**Median (IQR) uACR, mg/g**	**Key eligibility criteria**
**Type 2 diabetes at high risk of atherosclerotic cardiovascular disease**							
DECLARE-TIMI 58^18^ (dapagliflozin 10 mg)	17 160	4·2	17 160 (100%)	1724 (10%)	85 (16)	13·1 (6·0–43·6)	Type 2 diabetes Age ≥40 years and history of coronary, cerebral, or peripheral vascular disease; or age ≥55 years in men or ≥60 years in women with at least one cardiovascular risk factor Creatinine clearance ≥60 mL/min
CANVAS Program^21^, ^26^, ^27^, ^28^, ^29^ (canagliflozin 100–300 mg)	10 142	2·4	10 142 (100%)	1461 (14%)	77 (21)	12·3 (6·7–42·1)	Type 2 diabetes History of coronary, cerebral, or peripheral vascular disease; or age >50 years with at least two cardiovascular risk factors eGFR ≥30 mL/min per 1·73 m^2^
VERTIS CV^19^, ^30^ (ertugliflozin 5 mg or 15 mg)	8246	3·0	8246 (100%)	1958 (24%)	76 (21)	19·0 (6·0–68·0)	Type 2 diabetes History of coronary, cerebral, or peripheral vascular disease eGFR ≥30 mL/min per 1·73 m^2^
EMPA-REG OUTCOME^31^, ^32^, ^33^ (empagliflozin 10 mg or 25 mg)	7020	3·1	7020 (100%)	706 (10%)	74 (21)	17·7 (7·1–72·5)	Type 2 diabetes History of coronary, cerebral, or peripheral vascular disease eGFR ≥30 mL/min per 1·73 m^2^
**Heart failure**							
DAPA-HF^34^, ^35^ (dapagliflozin 10 mg)	4744	1·5	2139 (45%)*	4744 (100%)	Overall: 66 (19) Diabetes: 63 (19) No diabetes: 68 (19)	NA	Symptomatic chronic heart failure (NYHA class II–IV) with LVEF ≤40% (ie, reduced ejection fraction) NT-proBNP ≥600 pg/mL eGFR ≥30 mL/min per 1·73 m^2^ Appropriate doses of medical therapy and use of medical devices
EMPEROR-REDUCED^11^, ^17^, ^36^, ^37^ (empagliflozin 10 mg)	3730	1·3	1856 (50%)	3730 (100%)	Overall: 62 (22) Diabetes: 61 (22) No diabetes: 63 (21)	22·1 (8·0–81·3)	Chronic heart failure (NYHA class II–IV) with LVEF ≤40% (ie, reduced ejection fraction) NT-proBNP above a defined threshold (stratified by LVEF) Appropriate doses of medical therapy and use of medical devices
EMPEROR-PRESERVED^3^, ^11^, ^38^ (empagliflozin 10 mg)	5988	2·2	2938 (49%)	5988 (100%)	Overall: 61 (20) Diabetes: 60 (21) No diabetes: 62 (19)	21·0 (8·0–71·6)	Symptomatic chronic heart failure (NYHA class II–IV) with LVEF >40% Echocardiographic evidence of structural heart disease or hospitalisation for heart failure in the last year NT-proBNP >300 pg/mL (or >900 pg/mL if in atrial fibrillation) eGFR ≥20 mL/min per 1·73 m^2^ No recent coronary event
DELIVER^4^ (dapagliflozin 10 mg)	6263	2·3	3150 (50%)†	6263 (100%)	Overall: 61 (19) Diabetes: 60 (20) No diabetes: 63 (19)	NA	Symptomatic heart failure (NYHA class II–IV) with LVEF >40% (ambulatory or hospitalised) Echocardiographic evidence of structural heart disease NT-proBNP ≥300 pg/mL (or ≥600 pg/mL if in atrial fibrillation)
SOLOIST-WHF^20^ (sotagliflozin 200–400 mg)	1222	0·8	1222 (100%)	1222 (100%)	51 (17)‡	NA	Hospitalised for heart failure requiring intravenous therapy (ie, a heart failure population with a wide range of LVEFs) Type 2 diabetes eGFR ≥30 mL/min per 1·73 m^2^ No recent coronary event
**Chronic kidney disease**							
CREDENCE^7^, ^39^, ^40^ (canagliflozin 100 mg)	4401	2·6	4401 (100%)	652 (15%)	56 (18)	927 (463–1833)	Type 2 diabetes eGFR 30–90 mL/min per 1·73 m^2^ uACR 300–5000 mg/g Stable maximally tolerated RAS blockade Excluded suspected non-diabetic kidney disease
SCORED^9^ (sotagliflozin 200–400 mg)	10 584	1·3	10 584 (100%)	3283 (31%)	44 (11)‡	74 (17–481)	Type 2 diabetes eGFR 25–60 mL/min per 1·73 m^2^ At least one cardiovascular risk factor
DAPA-CKD^8^, ^10^, ^22^, ^23^, ^41^, ^42^ (dapagliflozin 10 mg)	4304	2·4	2906 (68%)	468 (11%)	Overall: 43 (12) Diabetes: 44 (13) No diabetes: 42 (12)	949 (477–1885)	eGFR 25–75 mL/min per 1·73 m^2^ uACR 200–5000 mg/g Stable maximally tolerated RAS blockade, unless documented intolerance Excluded polycystic kidney disease, lupus nephritis, or anti-neutrophil cytoplasmic antibody-associated vasculitis
EMPA-KIDNEY^12^, ^13^ (empagliflozin 10 mg)	6609	2·0	3040 (46%)†	658 (10%)	Overall: 37 (14) Diabetes: 36 (13) No diabetes: 39 (15)	329 (49–1069)	eGFR 20–45 mL/min per 1·73 m^2^ or eGFR 45–90 mL/min per 1·73 m^2^ with uACR ≥200 mg/g at screening§ Clinically appropriate RAS blockade, unless not indicated or not tolerated Excluded polycystic kidney disease

Median follow-up is reported without IQR as these data were not always available. eGFR=estimated glomerular filtration rate. LVEF=left ventricular ejection fraction. NA=not available. NT-proBNP=N-terminal prohormone brain natriuretic peptide. NYHA=New York Heart Association. RAS=renin angiotensin system. uACR=urinary albumin:creatinine ratio.

* Includes patients with HbA_1c_ ≥6·5% at enrolment.

† Includes patients with HbA_1c_ ≥6·5% at baseline, or with history or prevalent use of a glucose-lowering agent; DELIVER had four participants with uncertain diabetes status who were excluded from all analyses; 68 patients in EMPA-KIDNEY had type 1 diabetes.

‡ The mean and SD were estimated from reported median and IQR.

§ 254 participants with an eGFR <20 mL/min per 1·73 m^2^ at their randomisation visit.

**Figure 1:**
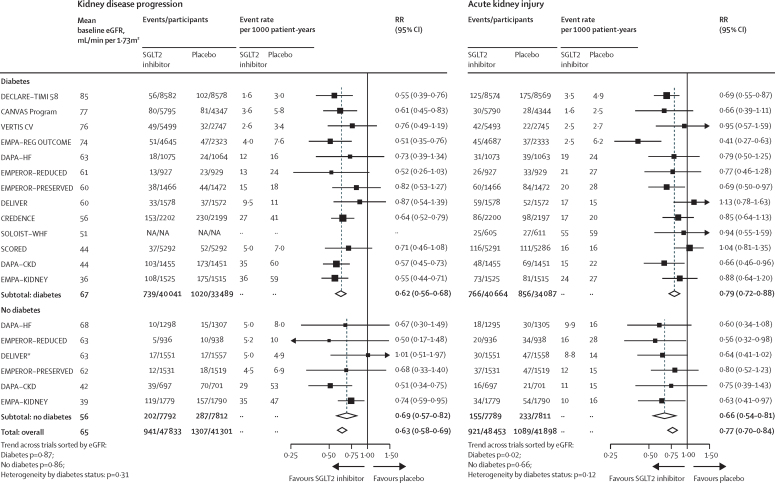
Effect of sodium glucose co-transporter-2 inhibition on kidney disease outcomes by diabetes status Kidney disease progression was defined as a sustained decrease in eGFR (≥50%) from randomisation, a sustained low eGFR, end-stage kidney disease, or death from kidney failure in all presented trials. Outcome definition details for each trial are provided in the appendix (pp 9–11). Rate values are not presented for the combined subtotal and total populations due to the heterogeneity in rates across the individual trials. eGFR=estimated glomerular filtration rate. RR=relative risk. SGLT2i=sodium glucose co-transporter-2 inhibitor. NA=not available. *One participant without diabetes in DELIVER was missing a baseline creatinine measurement and was excluded.

Compared with placebo, allocation to an SGLT2 inhibitor reduced the risk of kidney disease progression by 37% overall (RR 0·63, 95% CI 0·58–0·69; figure 1). The overall RR for the kidney failure subcomponent of this outcome in the chronic kidney disease trials was 0·67 (0·59–0·77, appendix p 15). For kidney disease progression, similar risk reductions were estimated in patients with diabetes (0·62, 0·56–0·68) and patients without diabetes (0·69, 0·57–0·82; heterogeneity p=0·31). There was no evidence that the RR reduction varied depending on mean baseline eGFR, either in those with diabetes (trend p=0·87) or those without diabetes (trend p=0·86; figure 1). Nor was there a significant trend in a sensitivity analysis in which trials were reordered by trial median baseline urine albumin-to-creatinine ratio (appendix p 16).

**Figure 2:**
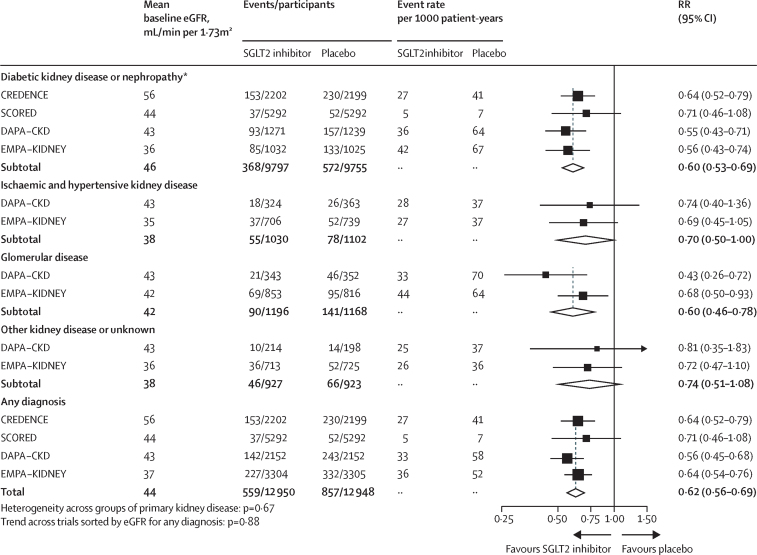
Effect of sodium glucose co-transporter-2 inhibition on kidney disease progression by presumed primary kidney disease (chronic kidney disease trials only) Effects in IgA nephropathy, focal segmental glomerulosclerosis, and other glomerular diseases considered separately are provided in the appendix (p 18). Rate values are not presented for the combined subtotal and total populations due to the heterogeneity in rates across the individual trials. eGFR=estimated glomerular filtration rate. RR=relative risk. SGLT2i=sodium glucose co-transporter-2 inhibitor. *RR in the diabetic kidney disease or nephropathy subgroup excluding SCORED (which did not formally assess primary kidney disease) is 0·59 (95% CI 0·52–0·68).

In the four chronic kidney disease trials, the RRs for kidney disease progression were similar when analyses were separated by primary kidney diagnosis (figure 2). In all four trials including patients with diabetic kidney disease, SGLT2 inhibitors reduced the risk of kidney disease progression by 40% (0·60, 0·53–0·69). Data from patients with non-diabetic causes of chronic kidney disease were available from the DAPA-CKD and EMPA-KIDNEY trials. SGLT2 inhibitors reduced the risk of kidney disease progression by 30% (0·70, 0·50–1·00) in patients with ischaemic and hypertensive kidney disease, by 40% (0·60, 0·46–0·78) in patients with glomerular diseases, and by 26% (0·74, 0·51–1·08) in patients with other or unknown causes combined, although 95% CIs were wide. When glomerular diseases were further divided into disease subcategories, we found no evidence of heterogeneity between patients with IgA nephropathy, focal segmental glomerulosclerosis, or other glomerulonephritis (appendix p 18).

Data on reported acute kidney injury were available from all included trials (appendix pp 10–11). Compared with placebo, allocation to an SGLT2 inhibitor reduced the risk of acute kidney injury by 23% overall (RR 0·77, 95% CI 0·70–0·84), with similar reductions observed in patients with diabetes (0·79, 0·72–0·88) and patients without diabetes (0·66, 0·54–0·81; heterogeneity p=0·12; figure 1). We found no strong evidence for differences in the relative effects by mean baseline eGFR (trend p=0·02 in patients with diabetes and p=0·66 in patients without diabetes; figure 1).

**Figure 3:**
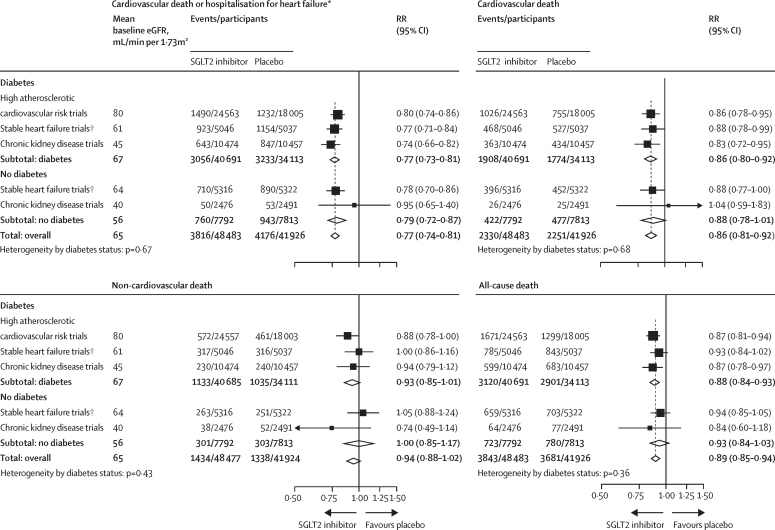
Effect of sodium glucose co-transporter-2 inhibition on heart failure and mortality outcomes by diabetes status Outcome data sources by trial are provided in the appendix (pp 10–11). Effects on heart failure and mortality were also analysed by trial with event rate per 1000 patient-years presented for each trial (appendix pp 19–20). eGFR=estimated glomerular filtration rate. RR=relative risk. SGLT2i=sodium glucose co-transporter-2 inhibitor. *Cardiovascular death or hospitalisation for heart failure outcomes exclude urgent heart failure visits. †Data from SOLOIST-WHF are included in totals but excluded from the stable heart failure trials group as the trial included patients with recent acute decompensated heart failure.

Overall, compared with placebo, allocation to an SGLT2 inhibitor reduced the risk of the composite outcome of cardiovascular death or hospitalisation for heart failure by 23% (RR 0·77, 95% CI 0·74–0·81; figure 3). The RRs were similar irrespective of a history of diabetes (0·77, 0·73–0·81 in patients with diabetes and 0·79, 0·72–0·87 in those without diabetes; heterogeneity p=0·67; figure 3, appendix p 19). Allocation to an SGLT2 inhibitor reduced the risk of cardiovascular death by 14% (0·86, 0·81–0·92), again with similar effects observed in those with diabetes (0·86, 0·80–0·92) and those without diabetes (0·88, 0·78–1·01; heterogeneity p=0·68). Allocation to an SGLT2 inhibitor did not significantly reduce the risk of non-cardiovascular death (0·94, 0·88–1·02), with similar RRs in patients with or without diabetes. The effects on heart failure or death did not appear to vary when trial results were ordered by mean baseline eGFR (appendix pp 19–20).

**Figure 4:**
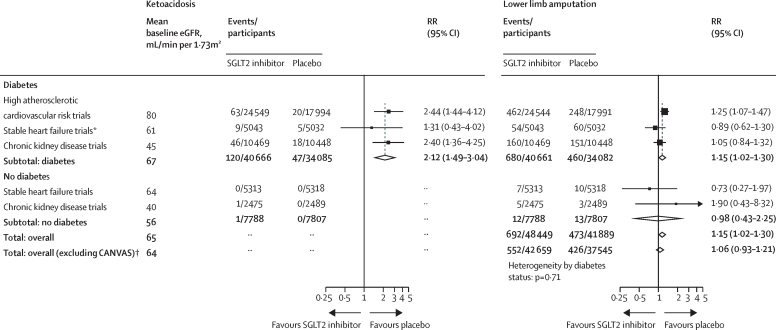
Effect of sodium glucose co-transporter-2 inhibition on ketoacidosis and lower limb amputation by diabetes status Effects on ketoacidosis and lower limb amputation were also analysed by trial with event rate per 1000 patient-years presented for each trial (appendix pp 21–22). Total values and forest plots are not presented for ketoacidosis due to the small number of events in patients without diabetes. eGFR=estimated glomerular filtration rate. RR=relative risk. SGLT2i=sodium glucose co-transporter-2 inhibitor. *Data from SOLOIST-WHF are included in totals but excluded from the stable heart failure trials group as the trial included patients with acute decompensated heart failure. †The hypothesis that SGLT2i might increase the risk of lower limb amputation was first raised by results from the CANVAS trial;^21^ the subtotal excluding CANVAS therefore reflects the combined results from the independent set of hypothesis-testing trials.

In patients with diabetes, the absolute risk of ketoacidosis was low (around 0·2 events per 1000 patient-years in placebo groups; appendix p 21). The RR for ketoacidosis in patients with diabetes allocated to an SGLT2 inhibitor, compared with placebo, was 2·12 (1·49–3·04; figure 4) and there was no evidence that this differed when trial results were ordered by mean baseline eGFR (appendix p 21). There was only one event of ketoacidosis among patients without diabetes receiving SGLT2 inhibitor during approximately 30 000 participant-years of follow-up.

In the CANVAS trial, allocation to an SGLT2 inhibitor was associated with a doubling in risk of lower limb amputation compared with placebo (6·3 *vs* 3·4 events per 1000 patient-years; appendix p 22). However in the other 12 trials, allocation to an SGLT2 inhibitor was not significantly associated with lower limb amputation (RR 1·06, 95% CI 0·93–1·21; figure 4; heterogeneity for CANVAS *vs* other 12 trials, p=0·0007). Thus across all trials, allocation to an SGLT2 inhibitor was associated with a 15% increase in the risk of lower limb amputation (1·15, 1·02–1·30). Compared with patients with diabetes, the baseline absolute risk of lower limb amputation was markedly lower among patients without diabetes. The RRs for amputations did not appear to vary depending on mean baseline eGFR (appendix p 22). The effects of SGLT2 inhibition on urinary tract infection (1·08, 1·02–1·15), serious urinary tract infection (1·07, 0·90–1·27), mycotic genital infections (3·57, 3·14–4·06), severe hypoglycaemia (0·89, 0·80–0·98), and bone fracture (1·07, 0·99–1·14) are shown in the appendix (appendix p 23).

**Figure 5:**
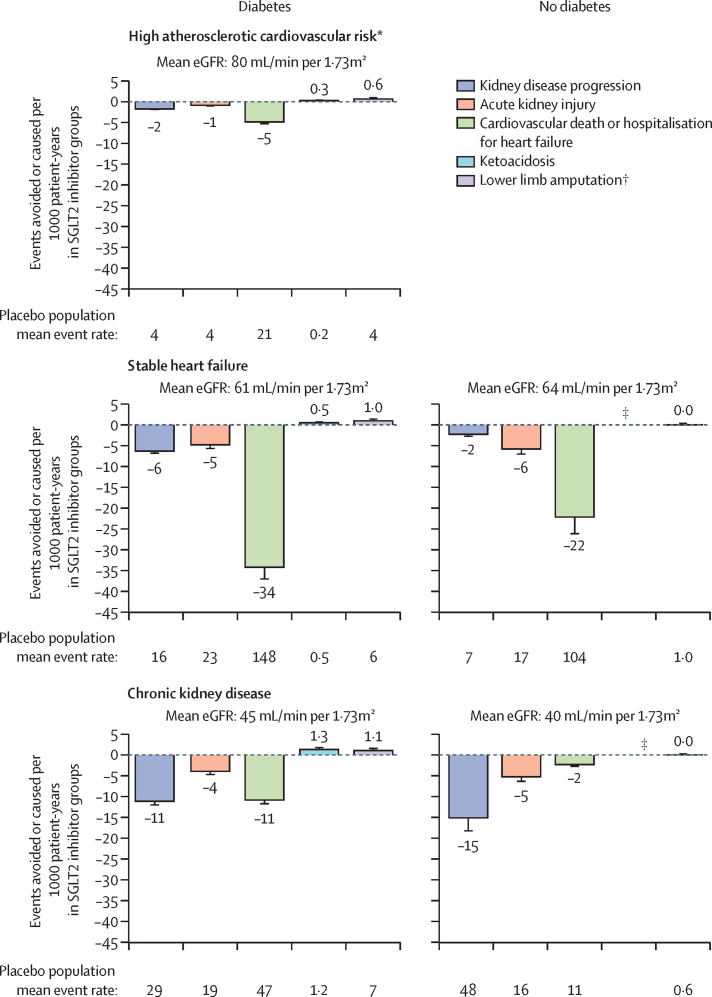
Absolute benefits and harms of SGLT2 inhibition per 1000 patient-years by diabetes status and patient group Patient group specific absolute effects estimated by applying the diabetes subgroup specific RR to the average event rate in the placebo arms (first event only). Negative numbers indicate events avoided by SGLT2 inhibition per 1000 patient-years. Error bars represent SE in the numbers of events avoided or caused, estimated from uncertainty in the RRs. Mean eGFR values are given for combined trial populations by patient group and diabetes status. Placebo population mean event rates are the absolute numbers of events per 1000 patient-years in the placebo groups of all trials in the relevant subpopulation. eGFR=estimated glomerular filtration rate. RR=relative risk. SGLT2i=sodium glucose co-transporter-2 inhibitor. *Additionally, two (SE 0·5) fewer myocardial infarctions per 1000 patient-years of SGLT2i treatment were observed in the diabetes and high atherosclerotic cardiovascular risk group. †RRs to determine absolute effects for lower limb amputation included CANVAS. ‡Too few ketoacidosis events to estimate absolute effects.

We estimated absolute rates and subsequently the benefits and harms of allocation to an SGLT2 inhibitor versus placebo, by diabetes status and by type of trial population (figure 5). In the studied participants, the absolute baseline risks of kidney disease progression, acute kidney injury, and cardiovascular death or hospitalisation for heart failure were generally slightly higher in patients with diabetes than in patients without diabetes. Consequently, in both participants with chronic kidney disease and participants with heart failure, the absolute benefits of SGLT2 inhibitor treatment were often larger for patients with diabetes. For example, treatment for one year of 1000 patients with chronic kidney disease and diabetes with an SGLT2 inhibitor was estimated to result in 11 fewer patients developing kidney disease progression, four fewer patients with acute kidney injury, and 11 fewer cardiovascular deaths or hospitalisations for heart failure, and to cause around one episode of ketoacidosis and around one lower limb amputation (figure 5). The corresponding benefits in patients with chronic kidney disease without diabetes were 15 fewer patients with kidney disease progression, five fewer with acute kidney injury, and two fewer cardiovascular deaths or hospitalisations for heart failure per 1000 patient-years, with no excess risk of ketoacidosis or amputation. In patients with heart failure, absolute benefits of SGLT2 inhibitor treatment on the outcome of cardiovascular death or hospitalisation for heart failure were notably large, irrespective of diabetes status (figure 5).

## Discussion

Large placebo-controlled trials of SGLT2 inhibitors have assessed patients with type 2 diabetes, chronic kidney disease, and heart failure, but no previous trial has been specifically powered to assess kidney or cardiovascular effects in patients without diabetes. Our key objective was to perform a collaborative meta-analysis incorporating all of the available evidence from all large SGLT2 inhibitor trials in populations with chronic kidney disease, heart failure, and type 2 diabetes at high cardiovascular risk, to compare the effects of SGLT2 inhibitors on the risk of kidney disease progression, acute kidney injury, and other key outcomes in patients with and without diabetes. Our analyses included information from around 90 000 trial participants, including about 16 000 people without diabetes. We defined kidney disease progression on the basis of a sustained decrease in eGFR (≥50%) from randomisation, a need to start maintenance dialysis or receive a kidney transplant, sustained low eGFR, or death from kidney failure. Our results showed that SGLT2 inhibitors reduce the risk of kidney disease progression by 37% and acute kidney injury by 23%, with similar effects in patients with and without diabetes. Patients with a wide range of kidney function have been studied in the reported trials, and despite attenuation of the effects of SGLT2 inhibitors on glycosuria with lower kidney function,^47^ our results did not suggest that kidney benefits were attenuated when trials were ordered by average baseline kidney function. SGLT2 inhibitors also appear safe at low levels of kidney function down to an eGFR of at least 20 mL/min per 1·73 m^2^ with patients without diabetes being at particularly low risk of ketoacidosis or amputation (whether receiving an SGLT2 inhibitor or not). In all the trial populations studied to date, the absolute benefits of SGLT2 inhibition considerably outweighed any serious hazards.

The outcome of a sustained decrease in eGFR (≥50%) from randomisation has been widely used to explore effects on kidney disease progression in subanalyses of the DAPA-CKD trial.^1^, ^8^, ^10^, ^22^, ^23^ This definition appears to be more specific for progression to kidney failure than lower thresholds for sustained decreases in eGFR (eg, ≥30% or ≥40%) when assessing interventions with a negative acute dip effect on eGFR, such as SGLT2 inhibitors. 48, 49, 50 The optimal percentage decrease in eGFR used to assess kidney disease progression is a trade-off between specificity (increased by larger percentage decreases) and outcome event rate (increased by smaller percentage decreases). DAPA-CKD suggested the effects of dapagliflozin on kidney disease progression were similar when participants with diabetic kidney disease or nephropathy, glomerular diseases, ischaemic or hypertensive kidney disease, and chronic kidney disease of other or unknown causes were considered separately.^10^ Furthermore, the DAPA-CKD investigators have reported results for 270 patients with IgA nephropathy, the commonest cause of glomerulonephritis worldwide, and reported kidney benefits in this particular subgroup (based on 25 kidney disease progression events).^22^ Analyses from EMPA-KIDNEY included 817 patients with IgA nephropathy and 80 kidney disease progression outcomes (appendix p 18). The current meta-analysis shows that the benefits of SGLT2 inhibitors on kidney disease progression extend to patients irrespective of diabetes status and in patients with chronic kidney disease irrespective of their primary kidney diagnosis.

Based on the average risk in different trial populations, we estimated that for every 1000 patients with chronic kidney disease treated for one year with an SGLT2 inhibitor, 11 first kidney disease progression events would be prevented in patients with diabetes, and 15 would be prevented in patients without diabetes. In these patients, such treatment also appeared to result in an estimated four to five fewer acute kidney injury events in patients with and without diabetes. Individual trials have shown that kidney benefits translate into important reductions in the need for dialysis or kidney transplantation,^7^, ^8^ and the cardiovascular and kidney benefits appear to be cost-saving in diabetic chronic kidney disease.^51^ We found no good evidence that the kidney benefits were modified by the average level of kidney function studied in the trials. Importantly, efficacy and safety data from EMPA-KIDNEY and DAPA-CKD combined include information on nearly 3000 patients with an eGFR of 20–30 mL/min per 1·73 m^2^. A total of 489 kidney disease progression outcomes accrued in those with an eGFR less than 30 mL/min per 1·73 m^2^ in those two trials.^7^, ^8^, ^52^ Although some clinical practice guidelines have started recommending use of SGLT2 inhibitors in type 2 diabetes at eGFRs down to 20 mL/min per 1·73 m^2^ (based on grade B levels of evidence),^53^, ^54^ many other recommendations limit initiation to those with eGFR greater than 25 mL/min per 1·73 m^2^ or 30 mL/min per 1·73 m^2^.^55^, ^56^, ^57^ As patients with decreased eGFR are at the highest absolute risk of kidney disease progression,^58^ our findings should encourage the initiation of SGLT2 inhibitors in patients with chronic kidney disease down to an eGFR of 20 mL/min per 1·73 m^2^ with continued use below this level. Several hundred participants in the chronic kidney disease trials had an eGFR below 20 mL/min per 1·73 m^2^ at randomisation or during follow-up (eg, 254 participants in EMPA-KIDNEY at randomisation), providing indirect evidence to support initiation of SGLT2 inhibitors in selected patients with an eGFR less than 20 mL/min per 1·73 m^2^.

This meta-analysis has a number of strengths: it addresses the scarcity of a single standardised kidney disease progression outcome in previous meta-analyses, and takes into account all of the available large-scale randomised evidence (at the time of publication) from around 90 000 people recruited into 13 relevant SGLT2 inhibitor clinical trials. The inclusion of new EMPA-KIDNEY and DELIVER data has more than doubled the number of outcomes previously available for kidney disease progression in patients without diabetes.^1^ Nevertheless, some limitations remain. Firstly, we found low numbers of cardiovascular deaths and heart failure hospitalisations in patients with chronic kidney disease without diabetes. Secondly, adjudication of acute kidney injury was not performed in most trials. Thirdly, individual participant-level data from all the trials are not yet available, precluding detailed analyses of the rate of change of eGFR (an accepted surrogate of kidney disease progression).^59^ Such analyses might have sufficient power to assess effects of SGLT2 inhibitors in individuals with slowly progressive chronic kidney disease in whom data are limited (eg, patients with chronic kidney disease with low levels of albuminuria). Fourthly, the efficacy and safety of SGLT2 inhibitors in people with established kidney failure requiring dialysis or kidney transplant remains to be evaluated (eg, NCT05374291), and data are insufficient to assess the effects on kidney and cardiovascular clinical outcomes for patients with other kidney diagnoses excluded from the chronic kidney disease trials (eg, polycystic kidney disease) and for patients with type 1 diabetes (appendix p 8).^44^, ^60^ Finally, our absolute effect estimates are specific to the recruited trial populations. RRs tend to be more generalisable, and so, in routine clinical practice, absolute effects of SGLT2 inhibitors could be estimated for an individual by calculating their absolute risk for an event with an established risk score, and then applying the RRs for the relevant outcome from the present meta-analysis.

In conclusion, our meta-analysis of the available large placebo-controlled SGLT2 inhibitor trials has shown that in the studied populations, SGLT2 inhibitors safely reduce the risk of kidney disease progression, acute kidney injury, cardiovascular death, and hospitalisation for heart failure in patients with chronic kidney disease or heart failure, irrespective of diabetes status. The proportional benefits were similar in patients with and without diabetes and appeared to be evident across the wide range of kidney function studied. In the trials of patients with chronic kidney disease, we also found that the proportional benefits on kidney disease progression were similar across the range of primary kidney diagnoses studied. The data from these large trials therefore support a central role for SGLT2 inhibitors as a disease-modifying therapy for chronic kidney disease, irrespective of diabetes status, primary kidney diagnosis, or level of kidney function.

Correspondence to: Assoc Prof William Herrington, Medical Research Council Population Health Research Unit at the University of Oxford, Nuffield Department of Population Health, Oxford OX3 7LF, UK **will.herrington@ndph.ox.ac.uk**

### Writing committee

### SMART-C steering committee

## Data sharing

All analysed summary data were extracted from published sources that are publicly available or were requested from individual trials (and are provided in the presented tables and figures). For the purpose of open access, the authors have applied a Creative Commons Attribution (CC BY) licence to any Author-Accepted Manuscript version arising.

## Declaration of interests

NS, RH, KJM, AJR, SYAN, DZ, PJ, DP, MJL, CB, JRE, and WGH report institutional grant funding from Boehringer Ingelheim and Eli Lilly for the EMPA-KIDNEY trial. NS additionally reports institutional grant funding from Novo Nordisk. RH additionally reports institutional grant funding from Novartis; and trial drug supply from Roche and Regeneron. CB additionally reports grant funding from the UK Medical Research Council, the UK National Institute for Health and Care Research Health Technology Assessment, and Health Data Research UK; and advisory roles for Merck, the National Institute for Health and Care Research Health Technology Assessment, the British Heart Foundation, and the European Society of Cardiology. WGH additionally reports funding from the UK Medical Research Council–Kidney Research UK Professor David Kerr Clinician Scientist Award. BLN reports consultancy fees and honorarium paid to his institution by AstraZeneca, Bayer, Boehringer Ingelheim, Cambridge Healthcare Research, American Diabetes Association, Renal Society of Australasia and Janssen; and advisory board membership (fees paid to institution) with AstraZeneca, Bayer, and Boehringer Ingelheim. SJH and MB are full-time employees of Boehringer Ingelheim International. SDA reports institutional grant funding from Vifor Int and Abbott Vascular; consultancy or advisory board fees from CVRx, Amgen, Respicardia, Novo Nordisk, Brahms, Novartis, Sanofi, and Cordio; and additional leadership or advisory board roles with Vifor Int, Bayer, Boehringer Ingelheim, Servier, Abbott Vascular, Impulse Dynamics, AstraZeneca, Bioventrix, Janssen, Cardior, V-Wave, Cardiac Dimensions, and Occlutech. JB reports consultancy fees and honorarium from Abbott, Adrenomed, Amgen, Applied Therapeutics, Array, AstraZeneca, Bayer, Boehringer Ingelheim, Bristol Myers Squibb, CVRx, G3 Pharma, Impulse Dynamics, Innolife, Janssen, LivaNova, Luitpold, Medtronic, Merck, Novartis, Novo Nordisk, Relypsa, Roche, Sequana Medical, and Vifor. DZIC reports institutional grant funding from Boehringer Ingelheim-Lilly, Merck, Janssen, Sanofi, AstraZeneca, CSL-Behring, and Novo Nordisk; and consultancy fees and honorarium from Boehringer Ingelheim-Lilly, Merck, AstraZeneca, Sanofi, Mitsubishi-Tanabe, AbbVie, Janssen, Bayer, Prometic, Bristol Myers Squibb, Maze, Gilead, CSL-Behring, Otsuka, Novartis, Youngene, Lexicon, and Novo Nordisk. JBG reports institutional grant funding from Boehringer Ingelheim-Lilly, Merck, Roche, and Sanofi and Lexicon; and consultancy fees from Boehringer Ingelheim-Lilly, Bayer, AstraZeneca, Sanofi and Lexicon, Hawthorne Effect and Omada, Pfizer, Valo, Anji, Vertex, and Novo Nordisk. C-CL is an employee of Merck Sharp & Dohme (a subsidiary of Merck & Co) and owns stock and/or stock options in Merck & Co. FRMcC reports grant funding from NIDDK, Satellite Healthcare, Advanced Medical, and Fifth Eye; and consultancy fees from GlaxoSmithKline, Advanced Medical, and Zydus Therapeutics. DKMcG reports consultancy fees from Merck & Co, Applied Therapeutics, Metavant, Sanofi, Afimmune, Lilly USA, Boehringer Ingelheim, Novo Nordisk, Bayer, GlaxoSmithKline, Lexicon, Altimmune, and Esperion; and other honorarium from Kirkland & Ellis, Pfizer, GlaxoSmithKline, Janssen, Afimmune, Sanofi, Boehringer Ingelheim, Merck & Co, AstraZeneca, Novo Nordisk, Esperion, and Lilly USA. JJVMcM reports institutional grant funding from AstraZeneca; consultancy fees from Abbott, Alkem Metabolics, Eris Lifesciences, Hikma, Lupin, Sun Pharmaceuticals, Heart.Org (Medscape Cardiology), ProAdWise Communications, Radcliffe Cardiology, Servier, and The Corpus; and fees paid to his institution for other advisory roles by Cytokinetics, Amgen, AstraZeneca, Theracos, Ionis Pharmaceuticals, DalCor, Cardurion, Novartis, GlaxoSmithKline, Bayer, KBP Biosciences, Boehringer Ingelheim, and Bristol Myers Squibb. MP reports personal fees from AbbVie, Actavis, Amarin, Amgen, AstraZeneca, Boehringer Ingelheim, Caladrius, Casana, CSL Behring, Cytokinetics, Imara, Lilly, Moderna, Novartis, Reata, Relypsa, and Salamandra. VP reports consultancy fees, honorarium, or advisory roles supported by AbbVie, Bayer, Boehringer Ingelheim, Chinook, GlaxoSmithKline, Janssen, Pfizer, AstraZeneca, Baxter, Eli Lilly, Gilead, Merck, Mitsubishi Tanabe, Mundipharma, Novartis, Novo Nordisk, Otsuka, Retrophin, Roche, Sanofi, Servier, and Vitae. MSS reports institutional grant funding from Abbott, Amgen, Anthos Therapeutics, AstraZeneca, Bayer, Daiichi-Sankyo, Eisai, Intarcia, Ionis, Medicines Company, MedImmune, Merck, Novartis, Pfizer, and Quark Pharmaceuticals; and consultancy fees from Althera, Amgen, Anthos Therapeutics, AstraZeneca, Beren Therapeutics, Bristol Myers Squibb, and DalCor. SDS reports institutional grant funding from Actelion, Alnylam, Amgen, AstraZeneca, Bellerophon, Bayer, BMS, Celladon, Cytokinetics, Eidos, Gilead, GSK, Ionis, Lilly, Mesoblast, MyoKardia, the National Heart, Lung, and Blood Institute (US National Institutes of Health), Neurotronik, Novartis, Novo Nordisk, Respicardia, Sanofi Pasteur, Theracos, and US2.AI; and consultancy fees from Abbott, Action, Akros, Alnylam, Amgen, Arena, AstraZeneca, Bayer, Boehringer Ingelheim, Bristol Myers Squibb, Cardior, Cardurion, Corvia, Cytokinetics, Daiichi-Sankyo, GlaxoSmithKline, Lilly, Merck, Myokardia, Novartis, Roche, Theracos, Quantum Genomics, Cardurion, Janssen, Cardiac Dimensions, Tenaya, Sanofi-Pasteur, Dinaqor, Tremeau, CellProThera, Moderna, American Regent, Sarepta, Lexicon, Anacardio, Akros, and Puretech Health. MV reports grant funding or advisory board fees from Amgen, AstraZeneca, American Regent, Baxter HealthCare, Bayer, Boehringer Ingelheim, Cytokinetics, Pharmacosmos, Relypsa, Novartis, Roche Diagnostics, Lexicon Pharmaceuticals, Galmed, Occlutech, Impulse Dynamics, Sanofi, and Tricog Health; speaker fees from AstraZeneca, Boehringer Ingelheim, Novartis, and Roche Diagnostics; and actively participates on clinical trial committees for studies sponsored by Galmed, Novartis, Bayer, Occlutech, and Impulse Dynamics. CW reports institutional grant funding from Boehringer Ingelheim; and consultancy fees and honorarium from Boehringer Ingelheim, AstraZeneca, Merck Sharp & Dohme, and Bayer. SDW reports institutional grant funding from Abbott, Amgen, Anthos Therapeutics, ARCA Biopharma, AstraZeneca, Bayer HealthCare Pharmaceuticals, Daiichi-Sankyo, Eisai, Intarcia, Ionis Pharmaceuticals, Janssen Research and Development, MedImmune, Merck, Novartis, Pfizer, Quark Pharmaceuticals, Regeneron Pharmaceuticals, Roche, Siemens Healthcare Diagnostics, Softcell Medical, The Medicines Company, and Zora Biosciences; and consultancy fees from AstraZeneca, Boston Clinical Research Institute, Icon Clinical, and Novo Nordisk. FZ reports consultancy fees from Amgen, Applied therapeutics, AstraZeneca, Bayer, Boehringer Ingelheim, Cardior, Cereno Scientific, CEVA, Cellprothera, CVRx, Novartis, Novo Nordisk, Servier, Merck, Bristol Myers Squibb; and honorarium or other personal fees from Boehringer Ingelheim, Merck, Bayer, Vifor, Fresenius, Roche Diagnostics, Hogan and Lovells, and Acceleron. HJLH reports grant funding from AstraZeneca, Boehringer Ingelheim, Janssen, and Novo Nordisk; consultancy fees from AstraZeneca, AbbVie, Bayer, Boehringer Ingelheim, CSL Behring, Chinook, Dimerix, Eli Lilly, Gilead, Goldfinch Bio, Merck, Novartis, Novo Nordisk, Janssen, and Travere Therapeutics; and other payment or honorarium from AstraZeneca, Novo Nordisk, and Eli Lilly. MJL additionally reports institutional grant funding from Novartis and Janssen; and trial drug supply from Roche and Regeneron.
