# Supplementary material for: Impact of diabetes on the effects of sodium glucose co-transporter-2 inhibitors on kidney outcomes: collaborative meta-analysis of large placebo-controlled trials
Source: Lancet. 2022 Nov 19;400(10365):1788–801. doi: 10.1016/S0140-6736(22)02074-8 (PMC7613836; doi:10.1016/S0140-6736(22)02074-8)
Supplement: Supplementary appendix [file mmc1.pdf]

THE LANCET

Supplementary appendix

This appendix formed part of the original submission and has been peer reviewed. We post it as supplied by the authors.

Supplement to: The Nuffield Department of Population Health Renal Studies Group and the SGLT2 inhibitor Meta-Analysis Cardio-Renal Trialists' Consortium. Impact of diabetes on the effects of sodium glucose co-transporter-2 inhibitors on kidney outcomes: collaborative meta-analysis of large placebo-controlled trials. *Lancet* 2022; published online Nov 6. https://doi.org/10.1016/S0140-6736(22)02074-8.

WEB MATERIALS

IMPACT OF DIABETES ON THE EFFECTS OF SODIUM GLUCOSE CO-TRANSPORTER-2 (SGLT2) INHIBITORS ON KIDNEY OUTCOMES: COLLABORATIVE META-ANALYSIS OF LARGE PLACEBO-CONTROLLED TRIALS

This supplemental material has been provided by the authors

to give readers additional information about their work.

WEB METHODS

WEB REFERENCES

WEBTABLES

Webtable 1: Definition of kidney disease progression by trial

Webtable 2: Sources of analysis data in 13 included trials

Webtable 3: Additional baseline characteristics of participants in 13 included trials

Webtable 4: Risk of bias assessments

WEBFIGURES

Webfigure 1: Study selection

Webfigure 2: Effect of SGLT2 inhibitors on KIDNEY FAILURE, by diabetes status

Webfigure 3: Effect of SGLT2 inhibitors on KIDNEY DISEASE outcomes, by diabetes status and uACR

Webfigure 4: Effect of SGLT2 inhibitors on KIDNEY DISEASE outcomes, by diabetes status

Webfigure 5: Effect of SGLT2 inhibitors on KIDNEY DISEASE PROGRESSION, by different glomerular diseases

Webfigure 6: Effect of SGLT2 inhibition on CARDIOVASCULAR DEATH or HOSPITALISATION FOR HEART FAILURE, by diabetes status

Webfigure 7: Effect of SGLT2 inhibitors on CARDIOVASCULAR and NON-CARDIOVASCULAR DEATH, by diabetes status

Webfigure 8: Effect of SGLT2 inhibitors on KETOACIDOSIS, by diabetes status

Webfigure 9: Effect of SGLT2 inhibitors on LOWER LIMB AMPUTATION, by diabetes status

Webfigure 10: Effect of SGLT2 inhibitors on ADDITIONAL SAFETY outcomes

LIST OF COLLABORATORS

WEB METHODS

Literature search methods

This systematic review is an update of a previous systematic review and meta-analysis (PROSPERO ID: CRD42021240468) (1). The present review was registered on PROSPERO ahead of the updated database search (PROSPERO ID: CRD42022351618). The subsequent systematic search of MEDLINE and EMBASE databases was undertaken on 5th September 2022, limited to studies added to the database after 29th August 2021 (the date of completion of the previously published meta-analysis literature search). The search strategy utilised was identical to that of the prior systematic review and meta-analysis (with the exception of limits applied to dates) including the use of validated filters for randomised controlled trials (see below for full list of search terms). No language restriction was applied. Identified records were downloaded to a dedicated database, and titles and abstracts were screened for relevance and duplicates by a single study author (AJR), together with studies identified during the previous systematic review. Further full text screening was undertaken using a previously piloted spreadsheet against pre-specified inclusion criteria to identify studies for inclusion. The final database included primary trial publications and subsidiary peer-reviewed publications.

The inclusion criteria for this study included those of the previous meta-analysis, with an additional requirement (pre-specified in the PROSPERO-registered protocol) that studies should have a duration of greater than 6 months. The final inclusion criteria were as follows:

- Parallel-group randomised controlled trial in adults
- Randomisation of ≥1000 participants to an SGLT2 inhibitor (including combined SGLT1/2 inhibitors) versus placebo (required ≥500 participants in each group)
- Duration ≥6 months (additional inclusion criterion for updated systematic review)
- Reporting any of the pre-specified main efficacy outcomes and any of the pre-specified safety outcomes

Where relevant, multiple reports from the same study were collated using the study acronym or National Clinical Trials (NCT) database reference number.

Outcomes

Pre-specified outcomes analysed in the present study comprised the following:

- Kidney disease progression based on at least a 50% sustained decline in estimated glomerular filtration rate from baseline (Webtable 1)

- Acute kidney injury
- Hospitalisation for heart failure or cardiovascular death
- Cardiovascular death
- Any death
- Non-cardiovascular death
- Ketoacidosis
- Lower limb amputation

Outcomes were analysed separately by diabetes status.

Safety analyses evaluated in the previous meta-analysis were additionally analysed:

- Urinary tract infections
- Mycotic genital infections
- Fournier's gangrene
- Severe hypoglycaemia
- Bone fracture

Data extraction

For each identified trial, relevant results were identified from primary or subsidiary peer-reviewed publications and transcribed to dedicated spreadsheets by two authors (KJM, AJR), with discrepancies resolved by consensus discussion. Where potentially relevant data from identified trials were not available in the peer-reviewed literature, trial authors were contacted to request additional data.

For each trial, extracted data included main eligibility criteria; follow-up duration; relevant participant characteristics on the trial level (including proportion of patients with type 2 diabetes or heart failure, and average kidney function); number of events and participants for each arm in reported comparisons; event rate per 1000 patient-years in each arm; and hazard ratio and 95% confidence intervals for relevant comparisons, if reported. Where possible, data were extracted separately for patients with and without type 2 diabetes and, where reported, stratified by presumed primary kidney disease and glomerular disease (for chronic kidney disease [CKD] trials only). Trials were classified according to their primary inclusion criteria into three populations: type 2 diabetes at high risk of cardiovascular disease, stable heart failure (i.e. not in receipt of intravenous diuretic therapy), and CKD.

Risk of bias assessment

Risk of bias was assessed using Version 2 of the Cochrane risk-of-bias tool for randomized trials (ROB2) by two authors (KJM, AJR), independently and in duplicate.

Search Strategy

MEDLINE Search strategy

1	randomized controlled trial.pt.
2	controlled clinical trial.pt.
3	randomized.ab.
4	placebo.ab.
5	clinical trials as topic.sh.
6	randomly.ab.
7	trial.ti.
8	1 or 2 or 3 or 4 or 5 or 6 or 7
9	exp animals/ not humans.sh.
10	8 not 9
11	exp Sodium-Glucose Transporter 2 Inhibitors/
12	sglt2.tw.
13	sglt-2.tw.
14	exp Sodium-Glucose Transporter 2/
15	sodium-glucose transporter$.tw.
16	sodium-glucose co-transporter$.tw.
17	sodium-glucose cotransporter$.tw.
18	(canagliflozin$ or dapagliflozin$ or empagliflozin$ or ertugliflozin$ or ipragliflozin$ or luseogliflozin$ or remogliflozin$ or sergliflozin$ or sotagliflozin$ or tofogliflozin$).tw.
19	11 or 12 or 13 or 14 or 15 or 16 or 17 or 18
20	10 and 19

EMBASE Search strategy

1	Randomized controlled trial/
2	Controlled clinical study/
3	random$.ti,ab.
4	randomization/
5	intermethod comparison/
6	placebo.ti,ab.
7	(compare or compared or comparison).ti.
8	((evaluated or evaluate or evaluating or assessed or assess) and (compare or compared or comparing or comparison)).ab.
9	(open adj label).ti,ab.
10	((double or single or doubly or singly) adj (blind or blinded or blindly)).ti,ab.
11	double blind procedure/
12	parallel group$1.ti,ab.
13	(crossover or cross over).ti,ab.
14	((assign$ or match or matched or allocation) adj5 (alternate or group$1 or intervention$1 or patient$1 or subject$1 or participant$1)).ti,ab.
15	(assigned or allocated).ti,ab.
16	(controlled adj7 (study or design or trial)).ti,ab.
17	(volunteer or volunteers).ti,ab.
18	human experiment/
19	trial.ti.
20	1 or 2 or 3 or 4 or 5 or 6 or 7 or 8 or 9 or 10 or 11 or 12 or 13 or 14 or 15 or 16 or 17 or 18 or 19
21	(random$ adj sampl$ adj7 ("cross section$" or questionnaire$1 or survey$ or database$1)).ti,ab. not (comparative study/ or controlled study/ or randomi?ed controlled.ti,ab. or randomly assigned.ti,ab.)
22	Cross-sectional study/ not (randomized controlled trial/ or controlled clinical study/ or controlled study/ or randomi?ed controlled.ti,ab. or control group$1.ti,ab.)
23	(((case adj control$) and random$) not randomi?ed controlled).ti,ab.
24	(Systematic review not (trial or study)).ti.
25	(nonrandom$ not random$).ti,ab.
26	"Random field$".ti,ab.
27	(random cluster adj3 sampl$).ti,ab.
28	(review.ab. and review.pt.) not trial.ti.
29	"we searched".ab. and (review.ti. or review.pt.)
30	"update review".ab.
31	(databases adj4 searched).ab.
32	(rat or rats or mouse or mice or swine or porcine or murine or sheep or lambs or pigs or piglets or rabbit or rabbits or cat or cats or dog or dogs or cattle or bovine or monkey or monkeys or trout or marmoset$1).ti. and animal experiment/
33	Animal experiment/ not (human experiment/ or human/)

34	21 or 22 or 23 or 24 or 25 or 26 or 27 or 28 or 29 or 30 or 31 or 32 or 33
35	20 not 34
36	exp Sodium-Glucose Transporter 2 Inhibitors/
37	sglt2.tw.
38	sglt-2.tw.
39	exp Sodium-Glucose Transporter 2/
40	sodium-glucose transporter$.tw.
41	sodium-glucose co-transporter$.tw.
42	sodium-glucose cotransporter$.tw.
43	(canagliflozin$ or dapagliflozin$ or empagliflozin$ or ertugliflozin$ or ipragliflozin$ or luseogliflozin$ or remogliflozin$ or sergliflozin$ or sotagliflozin$ or tofogliflozin$).tw.
44	36 or 37 or 38 or 39 or 40 or 41 or 42 or 43

Searches were limited to dates ranging from 29th August 2021 to the date of the search.

Data from eligible trials not included in meta-analyses

Two randomised trials were identified in the prior systematic review but not included in meta-analysis due to short follow-up duration (<6 months) and study populations not consistent with the pre-defined populations (type 2 diabetes with high atherosclerotic cardiovascular disease risk, heart failure, and CKD) in the previous meta-analysis. Data from these trials are summarised here.

InTandem3* (2) enrolled a population with type 1 diabetes and therefore did not fit into one of the 3 pre-defined patient groups. Follow-up was also only 24 weeks precluding the reporting of a large number of relevant outcomes. Outcomes relevant to this meta-analysis are summarised in the table below:

	**Sotagliflozin (n=699)** **Participants with events**	**Placebo (n=703)** **Participants with events**
**Hospitalisation for heart failure or cardiovascular death**	0	0
**"Renal event"**	5	3
**Ketoacidosis**	21	4
**Amputation**	0	0
**Any death**	1	0

* 24 week trial in a population with type 1 diabetes with primary outcome of: glycated haemoglobin level <7.0% at week 24, with no episodes of severe hypoglycaemia or diabetic ketoacidosis after randomisation.
Kidney disease progression and acute kidney injury events data not available.

The Dapagliflozin in Respiratory Failure in Patients With COVID-19 (DARE-19) trial (3) was identified by the systematic review but not included in meta-analysis given the study population (hospitalised patients with COVID-19) and very short treatment and follow-up duration (30 days), limiting reporting of most relevant outcomes. Reported outcomes relevant to this meta-analysis are summarised below:

	**Dapagliflozin (n=625)** **Participants with events**	**Placebo (n=625)** **Participants with events**
**Any death**	41	54
**Acute kidney injury**	21	34
**Diabetic ketoacidosis**	2	0

WEB REFERENCES

1. Staplin N, Roddick AJ, Emberson J, Reith C, Riding A, Wonnacott A, et al. Net effects of sodium-glucose co-transporter-2 inhibition in different patient groups: a meta-analysis of large placebo-controlled randomized trials. eClinicalMedicine. 2021;41.
2. Garg SK, Henry RR, Banks P, Buse JB, Davies MJ, Fulcher GR, et al. Effects of Sotagliflozin Added to Insulin in Patients with Type 1 Diabetes. New England Journal of Medicine. 2017;377(24):2337-48.
3. Kosiborod MN, Esterline R, Furtado RHM, Oscarsson J, Gasparyan SB, Koch GG, et al. Dapagliflozin in patients with cardiometabolic risk factors hospitalised with COVID-19 (DARE-19): a randomised, double-blind, placebo-controlled, phase 3 trial. Lancet Diabetes Endocrinol. 2021;9(9):586-94.

Webtable 1: Definition of kidney disease progression by trial

Trial	Components & definitions used in this meta-analysis					Definitions originally applied by individual trials
	Sustained ≥50% eGFR decline	Kidney replacement therapy	Sustained eGFR <15 (or <10)	Renal death	Definition of sustained	
DECLARE-TIMI 58	✓	✓	✓	✓	As confirmed by two tests at the central laboratory ≥4 weeks apart	Sustained ≥40% eGFR decline to <60 mL/min/1.73 m^2 or ESKD (defined as dialysis ≥90 days, kidney transplantation, or sustained eGFR <15 mL/min/1.73 m^2) or renal death
CANVAS Program	✓	✓	✓	✓	Two consecutive measurements ≥30 days apart unless identified on the last available measurement	Sustained 50% eGFR decline or ESKD (defined as maintenance dialysis ≥30 days, kidney transplantation, sustained eGFR <15 mL/min/1.73 m^2) or renal death
VERTIS CV	✓	✓	✓	✓	Subsequent value that also met the cut-off criterion >30 days later	Pre-specified secondary: Doubling of serum creatinine, dialysis*/kidney transplantation or renal death<br>Pre-specified exploratory: Sustained ≥40% decline in eGFR, chronic* dialysis/kidney transplantation or renal death
EMPA-REG OUTCOME	✓	✓	✓	✓	Sustained for ≥28 days according to central laboratory assessment	Pre-specified: Sustained ≥40% eGFR decline or ESKD (defined as "sustained continuous"* dialysis/ kidney transplantation or sustained eGFR <15 mL/min/1.73 m^2) or *hospitalisation for heart failure or cardiac* or renal *death*.<br>Post-hoc: Sustained ≥40%; (also published for ≥30%, ≥50% and ≥57%) eGFR decline or RRT initiation or renal death.
DAPA-HF	✓	✓	✓	✓	Defined as lasting ≥28 days	Sustained ≥50% eGFR decline, ESKD (defined as chronic* dialysis, kidney transplantation or sustained eGFR <15 mL/min/1.73 m^2) or renal death
EMPEROR-REDUCED	✓	✓	✓	✓	Sustained for ≥30 days according to central laboratory assessment or if the last measurement meets criteria and death occurred within 60 days	Sustained ≥40% eGFR decline or ESKD (defined as chronic* dialysis/ kidney transplantation or sustained eGFR <15 for patients with baseline eGFR ≥30, or sustained eGFR <10 for patients with baseline eGFR <30 mL/min/1.73 m^2)
EMPEROR-PRESERVED	✓	✓	✓	✓	Sustained for ≥30 days according to central laboratory assessment or if the last measurement meets criteria and death occurred within 60 days	Sustained ≥40% eGFR decline or ESKD (defined as chronic* dialysis/ kidney transplantation or sustained eGFR <15 for patients with baseline eGFR≥30 or sustained eGFR<10 for patients with baseline eGFR<30 mL/min/1.73 m^2)
DELIVER	✓	✓	✓	✓	Measured at two consecutive scheduled study follow-up visits (≥1 month apart), or at last available visit	Sustained ≥50% eGFR decline, ESKD (defined from adverse event reports), sustained eGFR <15 mL/min/1.73 m^2, or renal death
SOLOIST-WHF	Not available					
CREDENCE	✓	✓	✓	✓	Sustained for ≥30 days according to central laboratory assessment	Primary: Sustained doubling of serum creatinine, ESKD (defined as maintenance dialysis ≥30 days, kidney transplantation or sustained eGFR <15 mL/min/1.73 m^2) or renal or *cardiovascular death*<br>Secondary: Sustained doubling of serum creatinine, ESKD or renal death
SCORED	✓	✓	✓	X	Sustained for ≥30 days	Sustained ≥50% eGFR decline, long-term* dialysis, kidney transplantation or sustained eGFR <15 mL/min/1.73 m^2
DAPA-CKD	✓	✓	✓	✓	Two consecutive central laboratory eGFR values ≥28 days apart	Sustained ≥50% eGFR decline, ESKD (defined as maintenance dialysis ≥28 days, kidney transplantation or sustained eGFR <15 mL/min/1.73 m^2) or renal death
EMPA-KIDNEY	✓	✓	✓	✓	(a) measured at two consecutive scheduled study follow-up visits; or (b) last available measurement	Sustained ≥40% eGFR decline, ESKD (defined as maintenance dialysis ≥90 days or kidney transplantation), sustained eGFR <10 mL/min/1.73m² or renal death

Kidney disease progression definitions used for meta-analysis are provided in the left side of the table, with a record of original trial or post-hoc versions of the outcome provided in the final column on the right. * Duration undefined. eGFR=estimated glomerular filtration rate; ESKD=end-stage kidney disease. RRT=renal replacement therapy.

Webtable 2: Sources of analysis data in 13 included trials

Trial	Outcome	Provided after request	Published (required format)	Published (estimated indirectly)	Comment
**DECLARE-TIMI 58**	Kidney disease progression (≥50%)	✓			
	Any death, CV death; CV death/HHF		✓		
	Non-CV death		✓		
	AKI, amputation, ketoacidosis		✓	✓	Rates estimated from numbers of events. AKI: defined by MedDRA Preferred Term for AKI (serious and non-serious; unadjudicated).
	UTI, mycotic genital infections, hypoglycaemia, fractures			✓	Rates estimated from number of events. Serious UTI not reported.
**CANVAS Program**	Kidney disease progression (≥50%)		✓		Event numbers by arm estimated from reported event rates, total events and hazard ratios.
	Any death, CV death; CV death/HHF		✓	✓	Event numbers by arm estimated from reported event rates, total events and hazard ratios.
	Non-CV death			✓	Calculated indirectly from any death & CV death data.
	AKI, amputation, ketoacidosis		✓	✓	Risk ratio estimated from rates. Event numbers by arm estimated from reported event rates, total events and hazard ratios. AKI: defined by MedDRA Preferred Term for AKI (serious only; unadjudicated).
	UTI, mycotic genital infections, hypoglycaemia, fractures			✓	Risk ratio estimated from rates. Data for UTI and mycotic genital infections extracted from previous meta-analysis. Hypoglycaemia not included. Serious UTI not reported.
**VERTIS CV**	Kidney disease progression (≥50%)	✓			
	Any death, CV death; CV death/HHF		✓		
	Non-CV death			✓	Calculated indirectly from any death & CV death data.
	AKI, amputation, ketoacidosis		✓	✓	Rates & risk ratios estimated from event numbers. AKI: defined by MedDRA Preferred Term for AKI (serious only; unadjudicated).
	UTI, mycotic genital infections, hypoglycaemia, fractures			✓	Risk ratios and rates estimated from numbers of events. UTI and serious UTI reported separately.Mycotic genital infections: results for male and female infections combined by inverse-variance meta-analysis.
**EMPA-REG OUTCOME**	Kidney disease progression (≥50%)	✓			
	Any death, CV death; CV death/HHF		✓		CV death definition excluded stroke.
	Non-CV death			✓	Calculated indirectly from any death & CV death data.
	AKI, amputation, ketoacidosis		✓	✓	Amputation: published in required format. AKI & ketoacidosis: rates & risk ratios estimated from numbers of events. AKI: defined by MedDRA Preferred Term for AKI (serious and non-serious; unadjudicated).
	UTI, mycotic genital infections, hypoglycaemia, fractures			✓	Risk ratios and rates estimated from numbers of events. UTI and serious UTI reported separately.
**DAPA-HF**	Kidney disease progression (≥50%)		✓		
	Any death, CV death; CV death/HHF		✓		CV death/HHF: used analyses excluding urgent HF visits.
	Non-CV death			✓	Calculated indirectly from any death & CV death data.
	AKI, amputation, ketoacidosis	✓	✓	✓	AKI: provided as defined by MedDRA Preferred Term for AKI (serious and non-serious; unadjudicated). Amputation: Rates estimated from numbers of events. Ketoacidosis: Rates & risk ratios estimated from numbers of events.
	UTI, mycotic genital infections, hypoglycaemia, fractures			✓	Risk ratios and rates estimated from numbers of events. UTI and mycotic genital infections not reported.
**EMPEROR-REDUCED**	Kidney disease progression (≥50%)	✓			
	Any death, CV death; CV death/HHF		✓	✓	Any death: calculated indirectly rom CV and non-CV death
	Non-CV death	✓			
	AKI, amputation, ketoacidosis	✓	✓	✓	AKI: provided as defined by MedDRA Preferred Term for AKI (serious and non-serious; unadjudicated).Amputation: Risk ratios estimated from numbers of events. Ketoacidosis: zero events occurred in either treatment arm.
	UTI, mycotic genital infections, hypoglycaemia, fractures			✓	Risk ratios and rates estimated from numbers of events. UTI and serious UTI reported separately. Genital infections presented overall.
**EMPEROR-PRESERVED**	Kidney disease progression (≥50%)	✓			
	Any death, CV death; CV death/HHF		✓		CV death/HHF: rates estimated from numbers of events
	Non-CV death	✓			
	AKI, amputation, ketoacidosis	✓	✓	✓	AKI: provided as defined by MedDRA Preferred Term for AKI (serious and non-serious; unadjudicated).Amputation & ketoacidosis: Rates & risk ratios estimated from numbers of events.
	UTI, mycotic genital infections, hypoglycaemia, fractures			✓	Risk ratios and rates estimated from numbers of events. UTI and serious UTI reported separately. Genital infections presented overall. Hypoglycaemia includes non-severe hypoglycaemia.

Trial	Outcome	Provided after request	Published (required format)	Published (estimated indirectly)	Comment
**DELIVER**	Kidney disease progression (≥50%)	✓			Kidney function assessed at randomisation, and then at 1, 4, 12, 24 and 36 months.
	Any death; CV death; CV death/HHF	✓			
	Non-CV death			✓	Calculated indirectly from provided any death & CV death data.
	AKI, amputation, ketoacidosis	✓			AKI: defined by MedDRA Preferred Term for AKI (serious and non-serious; unadjudicated).
	UTI, mycotic genital infections, hypoglycaemia, fractures			✓	Risk ratios and rates estimated from numbers of events. UTI: includes serious adverse events only. Fracture and mycotic genital infections not reported.
**SOLOIST-WHF**	Kidney disease progression (≥50%)	NA	NA	NA	
	Any death, CV death; CV death/HHF		✓		CV death/HHF: Number of events & rates estimated. Used analyses excluding urgent HF visits.
	Non-CV death			✓	Calculated indirectly from any death & CV death data.
	AKI, amputation, ketoacidosis		✓	✓	Rates & risk ratios estimated from numbers of events. AKI: defined by MedDRA Preferred Term for AKI (treatment-emergent, serious and non-serious; unadjudicated).
	UTI, mycotic genital infections, hypoglycaemia, fractures			✓	Risk ratios and rates estimated from numbers of events. Serious UTI not reported. Mycotic genital infections: results for male and female infections combined by inverse-variance meta-analysis.
**CREDENCE**	Kidney disease progression (≥50%)	✓			
	Kidney failure		✓		
	Any death, CV death; CV death/HHF		✓		
	Non-CV death			✓	Calculated indirectly from any death & CV death data.
	AKI, amputation, ketoacidosis		✓		AKI: defined by MedDRA Preferred Term for AKI (serious and non-serious; unadjudicated).
	UTI, mycotic genital infections, hypoglycaemia, fractures			✓	Risk ratios and rates estimated from numbers of events. Serious UTI not reported. Mycotic genital infections: results for male and female infections combined by inverse-variance meta-analysis. Hypoglycaemia not limited to serious.
**SCORED**	Kidney disease progression (≥50%)		✓		
	Kidney failure	NA	NA	NA	
	Any death, CV death; CV death/HHF			✓	CV death/HHF: Rates estimated from numbers of events.
	Non-CV death			✓	Rates & risk ratio estimated from numbers of events.
	AKI, amputation, ketoacidosis			✓	AKI: event numbers estimated; defined by narrow SMQ for *acute renal failure* (serious and non-serious, unadjudicated). Amputation & ketoacidosis: rates & risk ratios estimated from numbers of events.
	UTI, mycotic genital infections, hypoglycaemia, fractures			✓	Risk ratios and rates estimated from numbers of events. Serious UTI not reported.
**DAPA-CKD**	Kidney disease progression (≥50%)		✓		
	Kidney failure		✓		
	Any death, CV death; CV death/HHF		✓		
	Non-CV death			✓	Calculated indirectly from any death & CV death data.
	AKI, amputation, ketoacidosis		✓		AKI: ascertained from outcome "abrupt decline in kidney function" (defined as a doubling of creatinine compared with most recent results; adjudicated).
	UTI, mycotic genital infections, hypoglycaemia, fractures			✓	Risk ratios and rates estimated from numbers of events. UTI and mycotic genital infections: includes serious adverse events only.
**EMPA-KIDNEY**	Kidney disease progression (≥50%)	✓			All data were previously unpublished and provided directly.
	Kidney failure	✓			All data were previously unpublished and provided directly.
	Any death; CV death; CV death/HHF	✓			All data were previously unpublished and provided directly.
	Non-CV death	✓			All data were previously unpublished and provided directly.
	AKI, amputation, ketoacidosis	✓			All data were previously unpublished and provided directly. AKI: adjudicated serious AKI based on standard definition of Serious Adverse Event.
	UTI, mycotic genital infections, hypoglycaemia, fractures	✓			All data were previously unpublished and provided directly. Risk ratios and rates estimated from numbers of events. UTI and mycotic genital infections: includes serious adverse events only.

Rates estimated as events per 1000 patient-years. Serious UTI includes events defined as serious as per ICH GCP criteria or events defined as complicated in primary trial publications. Kidney failure outcome applies only to CKD trials. Abbreviations: AKI = acute kidney injury; CV = cardiovascular; HHF = hospitalisation for heart failure; MedDRA = Medical Dictionary for Regulatory Activities; NA = not available from SOLOIST-WHF as data not collected; SMQ = Standardised MedDRA Query; UTI = urinary tract infection.

Webtable 3: Additional baseline characteristics of participants in 13 included trials

**Patient group** Trial acronym	**N**	**Mean (SD) age, years***	**Female sex, N (%)**	**RAS inhibitor, N (%)**	
				**ACE inhibitor**	**ARB**
**Type 2 diabetes at high ASCVD risk**					
DECLARE-TIMI 58	17160	63.9 (6.8)*	6422 (37.4)	13950 (81.3) †	
CANVAS Program	10142	63.3 (8.3)	3633 (35.8)	8116 (80.0) †	
VERTIS CV	8246	64.4 (8.1)	2477 (30.0)	6686 (81.1) †	
EMPA-REG OUTCOME	7020	63.1 (8.7)	2004 (28.5)	5666 (80.7) †	
**Heart failure**					
DAPA-HF	4744	66.3 (10.9)	1109 (23.4)	2661 (56.0)	1307 (27.6)
EMPEROR-REDUCED	3730	66.8 (11.0)	893 (23.9)	1703 (45.7)	908 (24.3)
EMPEROR-PRESERVED	5988	71.8 (9.5)	2676 (44.7)	2409 (40.2)	2316 (38.7)
DELIVER	6263	71.6 (9.6)	2747 (43.9)	2295 (36.6)	2272 (36.3)
SOLOIST-WHF	1222	69.7 (9.3)§	412 (33.7)	495 (40.5)	515 (42.1)
**Chronic kidney disease**					
CREDENCE	4401	63.0 (9.2)	1494 (33.9)	4395 (99.9) †	
SCORED	10584	68.7 (8.1)§	4754 (44.9)	4048 (38.2)	5181 (49.0)
DAPA-CKD	4304	61.9 (12.1)	1425 (33.1)	1354 (31.5)	2870 (66.7)
EMPA-KIDNEY	6609	63.9 (13.9)	2192 (33.2)	2211 (33.5)	3411 (51.6)

*Mean (SD) age calculated for overall cohort where reported only by treatment arm. †Not presented separately. §Mean (SD) estimated from median (IQR) reported by treatment arm. RAS = renin angiotensin system; ACE = angiotensin-converting enzyme; ARB = angiotensin receptor blocker.

Webtable 4: Risk of bias assessments

Study ID	Intervention	Comparator	Randomisation process	Deviations from the intended interventions	Missing outcome data	Measurement of the outcome	Selection of the reported result
DECLARE-TIMI 58	Dapagliflozin	Placebo	+	+	+	+	+
CANVAS Program	Canagliflozin	Placebo	+	+	+	+	+
VERTIS CV	Ertugliflozin	Placebo	+	+	+	+	+
EMPA-REG OUTCOME	Empagliflozin	Placebo	+	+	+	+	+
DAPA-HF	Dapagliflozin	Placebo	+	+	+	+	+
DELIVER	Dapagliflozin	Placebo	+	+	+	+	+
EMPEROR-REDUCED	Empagliflozin	Placebo	+	+	+	+	+
EMPEROR-PRESERVED	Empagliflozin	Placebo	+	+	+	+	+
CREDENCE	Canagliflozin	Placebo	+	+	+	+	+
SOLOIST-WHF	Sotagliflozin	Placebo	+	+	+	+	+
SCORED	Sotagliflozin	Placebo	+	+	+	+	+
DAPA-CKD	Dapagliflozin	Placebo	+	+	+	+	+
EMPA-KIDNEY	Empagliflozin	Placebo	+	+	+	+	+

Risk of bias of included trials as assessed using Version 2 of the Cochrane Risk-of-Bias tool for randomised trials (ROB2).

Key:

+	Low risk of bias
!	Some concerns
-	High risk of bias

Webfigure 1: Study selection

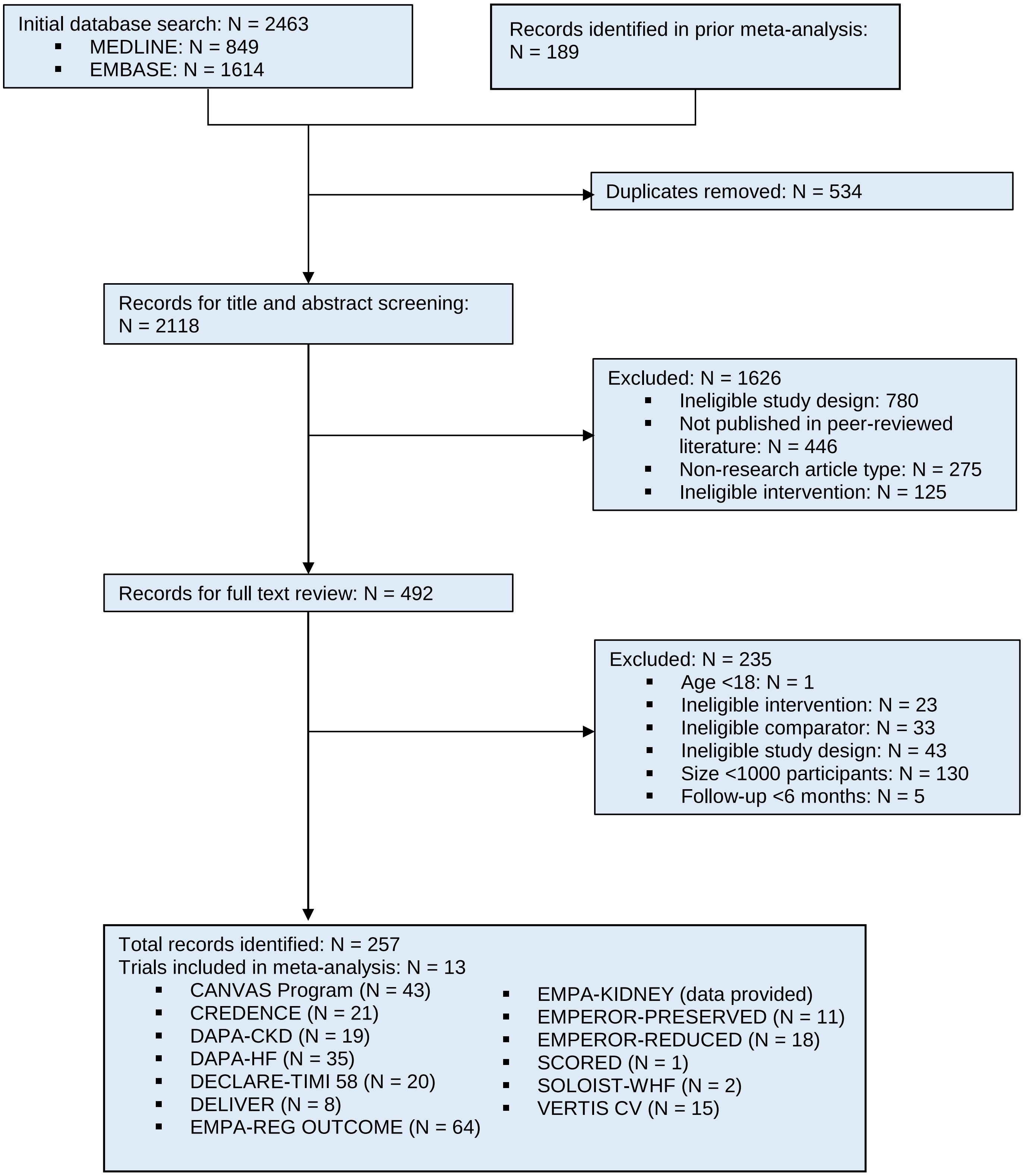

Multiple records were identified for many of the included trials. The breakdown of the 257 identified records by trial is listed in the final stage of the chart.

Webfigure 2: Effect of SGLT2 inhibition on KIDNEY FAILURE, by diabetes status (CKD trials only)

	Mean baseline eGFR (mL/min/1.73m²)	Events/participants SGLT2i	Events/participants Placebo	Rate per 1000 patient years SGLT2i	Rate per 1000 patient years Placebo	Relative risk (95% CI)	Trend across trials sorted by eGFR
**Diabetes**							
CREDENCE	56	116/2202	165/2199	20	29	0.68 (0.54, 0.86)	
SCORED	44	NA/NA	NA/NA				p=0.48
DAPA-CKD	44	77/1455	109/1451	26	37	0.69 (0.51, 0.92)	
EMPA-KIDNEY	36	74/1525	116/1515	24	39	0.59 (0.44, 0.79)	
**Subtotal: DIABETES**	**47**	**267/5182**	**390/5165**			**0.66 (0.56, 0.77)**	
**No diabetes**							
DAPA-CKD	42	32/697	52/701	24	39	0.56 (0.36, 0.87)	p=0.19
EMPA-KIDNEY	39	83/1779	105/1790	25	31	0.80 (0.60, 1.07)	
**Subtotal: NO DIABETES**	**40**	**115/2476**	**157/2491**			**0.72 (0.56, 0.91)**	
**TOTAL: OVERALL**	**45**	**382/7658**	**547/7656**			**0.67 (0.59, 0.77)**	

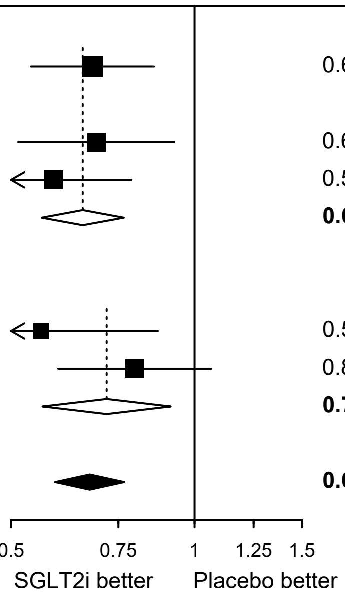

Heterogeneity by diabetes status: p=0.54

Kidney failure defined as composite of sustained eGFR<15 mL/min/1.73m² (or eGFR <10 mL/min/1.73m² in EMPA-KIDNEY), maintenance dialysis, or kidney transplantation. Data for kidney failure not available for SCORED. CI = confidence interval. eGFR = estimated glomerular filtration rate. SGLT2i = sodium-glucose co-transporter-2 inhibitor.

Webfigure 3: Effect of SGLT2 inhibitors on KIDNEY DISEASE outcomes, by diabetes status and uACR

	Median baseline uACR (mg/g)	KIDNEY DISEASE PROGRESSION: Number of events/participants		Rate per 1000 patient years: SGLT2i	Rate per 1000 patient years: Placebo	Relative risk (95% CI)	ACUTE KIDNEY INJURY: Number of events/participants		Rate per 1000 patient years: SGLT2i	Rate per 1000 patient years: Placebo	Relative risk (95% CI)
**Diabetes**											
CANVAS Program	12	80/5795	81/4347	3.6	5.8	0.61 (0.45, 0.83)	30/5790	28/4344	1.6	2.5	0.66 (0.39, 1.11)
DECLARE-TIMI 58	13	56/8582	102/8578	1.6	3.0	0.55 (0.39, 0.76)	125/8574	175/8569	3.5	4.9	0.69 (0.55, 0.87)
EMPA-REG OUTCOME	18	51/4645	47/2323	4.0	7.6	0.51 (0.35, 0.76)	45/4687	37/2333	2.5	6.2	0.41 (0.27, 0.63)
VERTIS CV	19	49/5499	32/2747	2.6	3.4	0.76 (0.49, 1.19)	42/5493	22/2745	2.5	2.7	0.95 (0.57, 1.59)
EMPEROR-PRESERVED	30	38/1466	44/1472	15	18	0.82 (0.53, 1.27)	60/1466	84/1472	20	28	0.69 (0.50, 0.97)
EMPEROR-REDUCED	35	13/927	23/929	13	24	0.52 (0.26, 1.03)	26/927	33/929	21	27	0.77 (0.46, 1.28)
SCORED	74	37/5292	52/5292	5.0	7.0	0.71 (0.46, 1.08)	116/5291	111/5286	16	16	1.04 (0.81, 1.35)
EMPA-KIDNEY	263	108/1525	175/1515	36	59	0.55 (0.44, 0.71)	73/1525	81/1515	24	27	0.88 (0.64, 1.20)
CREDENCE	927	153/2202	230/2199	27	41	0.64 (0.52, 0.79)	86/2200	98/2197	17	20	0.85 (0.64, 1.13)
DAPA-CKD	1017	103/1455	173/1451	35	60	0.57 (0.45, 0.73)	48/1455	69/1451	15	22	0.66 (0.46, 0.96)
DAPA-HF		18/1075	24/1064	12	16	0.73 (0.39, 1.34)	31/1073	39/1063	19	24	0.79 (0.50, 1.25)
SOLOIST-WHF		NA/NA	NA/NA				25/605	27/611	55	59	0.94 (0.55, 1.59)
DELIVER		33/1578	37/1572	9.5	11	0.87 (0.54, 1.39)	59/1578	52/1572	17	15	1.13 (0.78, 1.63)
**Subtotal: DIABETES**		**739/40041**	**1020/33489**			**0.62 (0.56, 0.68)**	**766/40664**	**856/34087**			**0.79 (0.72, 0.88)**
**No diabetes**											
EMPEROR-REDUCED	15	5/936	10/938	5.2	10	0.50 (0.17, 1.48)	20/936	34/938	16	28	0.56 (0.32, 0.98)
EMPEROR-PRESERVED	16	12/1531	18/1519	4.5	6.9	0.68 (0.33, 1.40)	37/1531	47/1519	12	15	0.80 (0.52, 1.23)
EMPA-KIDNEY	380	119/1779	157/1790	35	47	0.74 (0.59, 0.95)	34/1779	54/1790	10	16	0.63 (0.41, 0.97)
DAPA-CKD	861	39/697	70/701	29	53	0.51 (0.34, 0.75)	16/697	21/701	11	15	0.75 (0.39, 1.43)
DAPA-HF		10/1298	15/1307	5.0	8.0	0.67 (0.30, 1.49)	18/1295	30/1305	9.9	16	0.60 (0.34, 1.08)
DELIVER		17/1551	17/1557	5.0	4.9	1.01 (0.51, 1.97)	30/1551	47/1558	8.8	14	0.64 (0.41, 1.02)
**Subtotal: NO DIABETES**		**202/7792**	**287/7812**			**0.69 (0.57, 0.82)**	**155/7789**	**233/7811**			**0.66 (0.54, 0.81)**
**TOTAL: OVERALL**		**941/47833**	**1307/41301**			**0.63 (0.58, 0.69)**	**921/48453**	**1089/41898**			**0.77 (0.70, 0.84)**

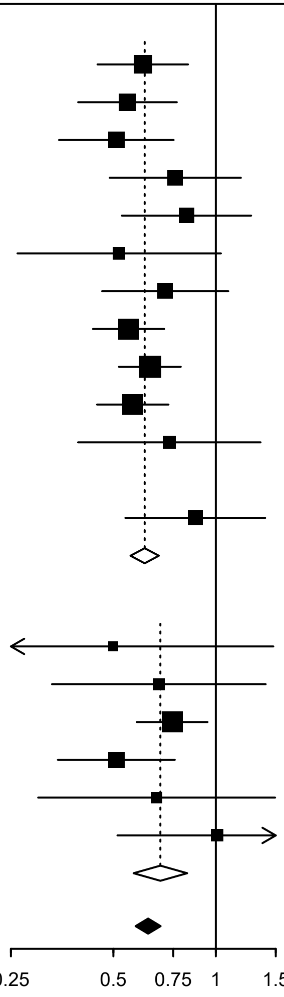

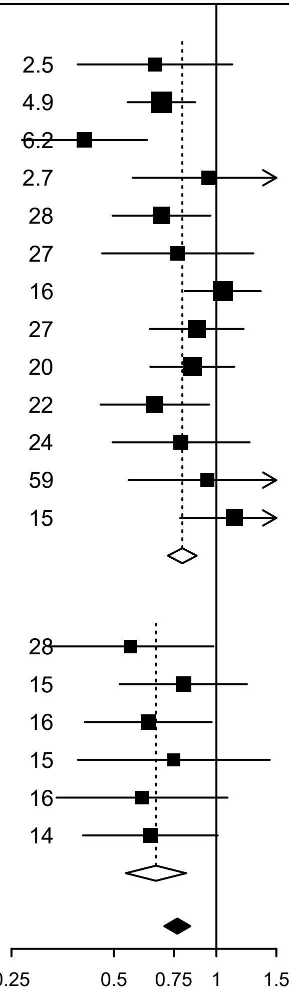

Kidney disease progression

Trend across trials sorted by uACR:
Diabetes p=1.00;
No diabetes p=0.47;

Heterogeneity by diabetes status: p=0.31

Acute kidney injury

Trend across trials sorted by uACR:
Diabetes p=0.05;
No diabetes p=0.75;

Heterogeneity by diabetes status: p=0.12

Kidney disease progression: analyses are based upon ≥50% decline in eGFR in all presented trials (see Webtable 1 for outcome definition details). Acute kidney injury definitions for each trial are provided in Webtable 2. Trials that did not report baseline uACR are excluded from the trend test. CI = confidence interval. eGFR = estimated glomerular filtration rate. SGLT2i = sodium-glucose co -transporter-2 inhibitor.

Webfigure 4: Effect of SGLT2 inhibitors on KIDNEY DISEASE outcomes, by diabetes status

	Mean baseline eGFR (mL/min/1.73m²)	KIDNEY DISEASE PROGRESSION: Events/participants SGLT2i	KIDNEY DISEASE PROGRESSION: Events/participants Placebo	KIDNEY DISEASE PROGRESSION: Relative risk (95% CI)	ACUTE KIDNEY INJURY: Events/participants SGLT2i	ACUTE KIDNEY INJURY: Events/participants Placebo	ACUTE KIDNEY INJURY: Relative risk (95% CI)
**Diabetes**							
High atherosclerotic CV risk trials	80	236/24521	262/17995	0.59 (0.49, 0.71)	242/24544	262/17991	0.65 (0.55, 0.78)
Stable heart failure trials	61	102/5046	128/5037	0.77 (0.59, 1.00)	176/5044	208/5036	0.83 (0.68, 1.02)
Chronic kidney disease trials	45	401/10474	630/10457	0.60 (0.53, 0.68)	323/10471	359/10449	0.88 (0.76, 1.02)
**Subtotal: DIABETES**	**67**	**739/40041**	**1020/33489**	**0.62 (0.56, 0.68)**	**766/40664**	**856/34087**	**0.79 (0.72, 0.88)**
**No diabetes**							
Stable heart failure trials	64	44/5316	60/5321	0.74 (0.50, 1.10)	105/5313	158/5320	0.66 (0.52, 0.85)
Chronic kidney disease trials	40	158/2476	227/2491	0.67 (0.55, 0.83)	50/2476	75/2491	0.67 (0.46, 0.95)
**Subtotal: NO DIABETES**	**56**	**202/7792**	**287/7812**	**0.69 (0.57, 0.82)**	**155/7789**	**233/7811**	**0.66 (0.54, 0.81)**
**TOTAL: OVERALL**	**65**	**941/47833**	**1307/41301**	**0.63 (0.58, 0.69)**	**921/48453**	**1089/41898**	**0.77 (0.70, 0.84)**

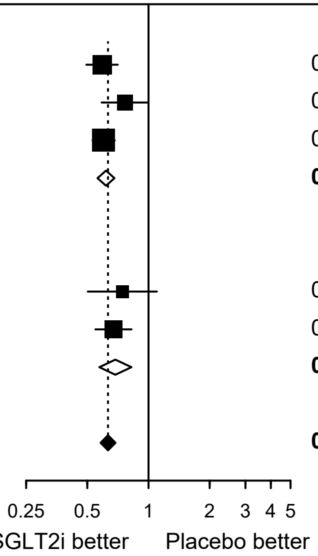

Heterogeneity by diabetes status: p=0.31

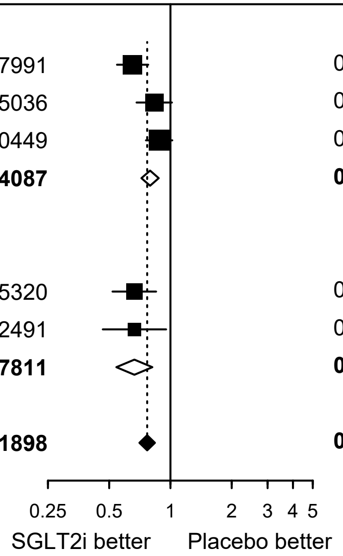

Heterogeneity by diabetes status: p=0.12

Kidney disease progression: analyses are based upon a sustained ≥50% decline in eGFR from randomisation, end-stage kidney disease or death from kidney failure in all presented trials (see Webtable 1 for outcome definition details). Acute kidney injury definitions for each trial are provided in Webtable 2. Data from SOLOIST-WHF excluded from the stable heart failure trials group as it included patients with acute decompensated heart failure. CI = confidence interval. eGFR = estimated glomerular filtration rate. SGLT2i = sodium-glucose co-transporter-2 inhibitor.

Webfigure 5: Effect of SGLT2 inhibitors on KIDNEY DISEASE PROGRESSION, by different glomerular diseases

	Average baseline eGFR (mL/min/1.73m²)	Events/participants		Rate per 1000 patient years		Relative risk (95% CI)
		SGLT2i	Placebo	SGLT2i	Placebo	
**IgA nephropathy**						
DAPA-CKD	44	5/137	20/133	21	88	0.24 (0.09, 0.65)
EMPA-KIDNEY	43	32/413	48/404	43	65	0.56 (0.36, 0.89)
**Subtotal**	**43**	**37/550**	**68/537**			**0.49 (0.32, 0.74)**
**Focal segmental glomerulosclerosis**						
DAPA-CKD	42	4/53	7/62	37	57	0.52 (0.15, 1.83)
EMPA-KIDNEY	41	9/98	8/97	46	43	1.24 (0.47, 3.25)
**Subtotal**	**41**	**13/151**	**15/159**			**0.89 (0.42, 1.92)**
**Other glomeronephritis**						
DAPA-CKD	43	12/153	19/157	33	50	0.65 (0.33, 1.29)
EMPA-KIDNEY	42	28/342	39/315	44	68	0.70 (0.43, 1.15)
**Subtotal**	**42**	**40/495**	**58/472**			**0.68 (0.46, 1.02)**
**ANY GLOMERULAR DISEASE**						
DAPA-CKD	43	21/343	46/352	33	70	0.43 (0.26, 0.72)
EMPA-KIDNEY	42	69/853	95/816	44	64	0.68 (0.50, 0.93)
**TOTAL**	**42**	**90/1196**	**141/1168**			**0.60 (0.46, 0.78)**

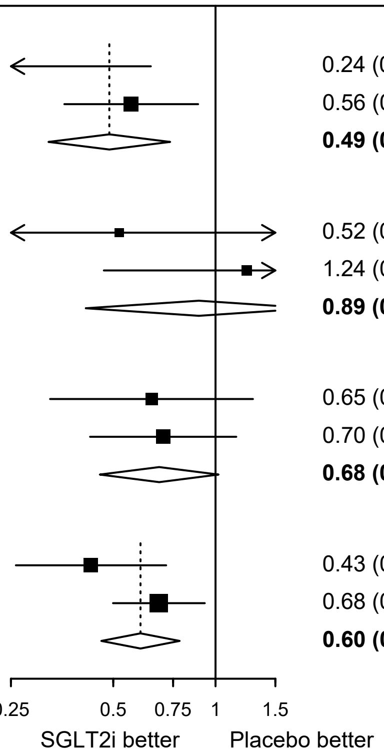

Heterogeneity across three subtypes of glomerular disease: p=0.30

Based on investigator-reported primary kidney diagnoses. CI = confidence interval. eGFR = estimated glomerular filtration rate. SGLT2i = sodium-glucose co-transporter-2 inhibitor.

Webfigure 6: Effect of SGLT2 inhibition on CARDIOVASCULAR DEATH or HOSPITALISATION FOR HEART FAILURE, by diabetes status

	Average baseline eGFR (mL/min/1.73m²)	Events/participants		Rate per 1000 patient years		Relative risk (95% CI)	Trend across trials sorted by eGFR
		SGLT2i	Placebo	SGLT2i	Placebo		
**Diabetes**							
DECLARE-TIMI 58	85	417/8582	496/8578	12	15	0.83 (0.73, 0.95)	
CANVAS Program	77	364/5795	288/4347	16	21	0.78 (0.67, 0.91)	
VERTIS CV	76	444/5499	250/2747	23	27	0.88 (0.75, 1.03)	
EMPA-REG OUTCOME	74	265/4687	198/2333	20	30	0.66 (0.55, 0.79)	
DAPA-HF	63	213/1075	268/1064	144	191	0.75 (0.63, 0.90)	
EMPEROR-REDUCED	61	200/927	265/929	177	246	0.72 (0.60, 0.87)	
EMPEROR-PRESERVED	60	239/1466	291/1472	83	102	0.79 (0.67, 0.94)	p=0.22
DELIVER	60	271/1578	330/1572	83	104	0.80 (0.68, 0.93)	
CREDENCE	56	179/2202	253/2199	32	45	0.69 (0.57, 0.83)	
SOLOIST-WHF	51	NA/608	NA/614	-	-	0.71 (0.56, 0.89)	
SCORED	44	283/5292	357/5292	41	52	0.77 (0.66, 0.91)	
DAPA-CKD	44	85/1455	119/1451	27	38	0.70 (0.53, 0.92)	
EMPA-KIDNEY	36	96/1525	118/1515	32	40	0.78 (0.60, 1.03)	
**Subtotal: DIABETES**	**67**	**3056/40691**	**3233/34113**			**0.77 (0.73, 0.81)**	
**No diabetes**							
DAPA-HF	68	169/1298	227/1307	91	124	0.73 (0.60, 0.89)	
EMPEROR-REDUCED	63	161/936	197/938	139	176	0.78 (0.64, 0.97)	
DELIVER	63	204/1551	246/1558	62	76	0.82 (0.68, 0.99)	
EMPEROR-PRESERVED	62	176/1531	220/1519	56	72	0.78 (0.64, 0.95)	p=0.28
DAPA-CKD	42	15/697	19/701	11	13	0.79 (0.40, 1.55)	
EMPA-KIDNEY	39	35/1779	34/1790	10	9.9	1.04 (0.65, 1.67)	
**Subtotal: NO DIABETES**	**56**	**760/7792**	**943/7813**			**0.79 (0.72, 0.87)**	
**TOTAL: OVERALL**	**65**	**3816/48483**	**4176/41926**			**0.77 (0.74, 0.81)**	

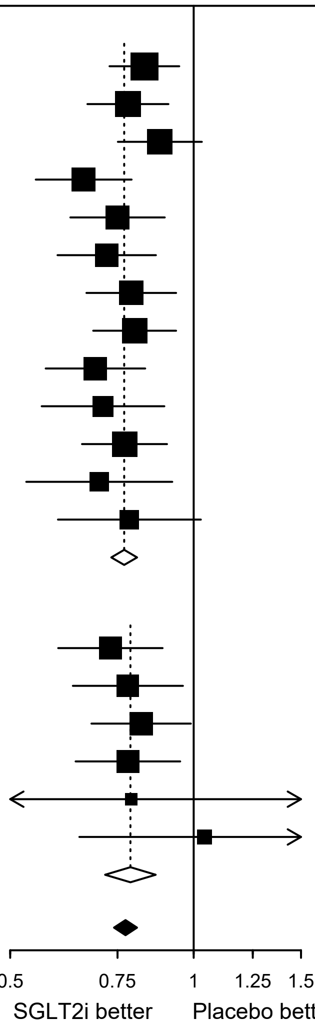

Heterogeneity by diabetes status: p=0.67

Excludes urgent heart failure visits. EMPA-REG OUTCOME cardiovascular death definition excluded stroke. CI = confidence interval. eGFR = estimated glomerular filtration rate. SGLT2i = sodium-glucose co-transporter-2 inhibitor.

Webfigure 7: Effect of SGLT2 inhibitors on CARDIOVASCULAR and NON-CARDIOVASCULAR DEATH, by diabetes status

	Mean baseline eGFR (mL/min/1.73m²)	CARDIOVASCULAR DEATH Events/participants SGLT2i	Events/participants Placebo	Rate per 1000 patient years SGLT2i	Rate per 1000 patient years Placebo	Relative risk (95% CI)	NON-CARDIOVASCULAR DEATH Events/participants SGLT2i	Events/participants Placebo	Rate per 1000 patient years SGLT2i	Rate per 1000 patient years Placebo	Relative risk (95% CI)
**Diabetes**											
DECLARE-TIMI 58	85	245/8582	249/8578	6.8	6.9	0.98 (0.82, 1.17)	211/8582	238/8578	5.9	6.6	0.88 (0.73, 1.06)
CANVAS Program	77	268/5795	185/4347	12	13	0.87 (0.72, 1.06)	132/5795	96/4347	5.7	6.7	0.87 (0.67, 1.13)
VERTIS CV	76	341/5499	184/2747	18	19	0.92 (0.77, 1.11)	132/5493	70/2745	6	7	0.94 (0.71, 1.25)
EMPA-REG OUTCOME	74	172/4687	137/2333	12	20	0.62 (0.49, 0.77)	97/4687	57/2333	7	8.4	0.85 (0.61, 1.17)
DAPA-HF	63	121/1075	148/1064	77	97	0.79 (0.63, 1.01)	22/1075	30/1064	14	20	0.73 (0.42, 1.25)
EMPEROR-REDUCED	61	104/927	113/929	84	91	0.92 (0.71, 1.20)	38/927	36/929	31	29	1.03 (0.65, 1.62)
EMPEROR-PRESERVED	60	120/1466	123/1472	39	39	0.99 (0.77, 1.27)	114/1466	103/1472	37	33	1.12 (0.86, 1.47)
DELIVER	60	123/1578	143/1572	35	41	0.85 (0.67, 1.08)	143/1578	147/1572	41	42	0.97 (0.78, 1.21)
CREDENCE	56	110/2202	140/2199	19	24	0.78 (0.61, 1.00)	58/2202	61/2199	10	11	0.94 (0.66, 1.35)
SOLOIST-WHF	51	51/608	58/614	106	125	0.84 (0.58, 1.22)	14/608	18/614	29	38	0.79 (0.39, 1.57)
SCORED	44	155/5292	170/5292	22	24	0.90 (0.73, 1.12)	85/5292	67/5292	13	11	1.20 (0.89, 1.62)
DAPA-CKD	44	56/1455	66/1451	17	21	0.85 (0.59, 1.21)	28/1455	47/1451	8	13	0.59 (0.37, 0.94)
EMPA-KIDNEY	36	42/1525	58/1515	14	19	0.71 (0.48, 1.06)	59/1525	65/1515	19	21	0.87 (0.61, 1.25)
**Subtotal: DIABETES**	**67**	**1908/40691**	**1774/34113**			**0.86 (0.80, 0.92)**	**1133/40685**	**1035/34111**			**0.93 (0.85, 1.01)**
**No diabetes**											
DAPA-HF	68	106/1298	125/1307	55	65	0.85 (0.66, 1.10)	27/1298	26/1307	14	13	1.05 (0.61, 1.78)
EMPEROR-REDUCED	63	83/936	89/938	67	72	0.92 (0.68, 1.24)	24/936	28/938	19	23	0.78 (0.45, 1.35)
DELIVER	63	108/1551	117/1558	31	34	0.93 (0.72, 1.21)	123/1551	117/1558	36	34	1.06 (0.83, 1.35)
EMPEROR-PRESERVED	62	99/1531	121/1519	30	37	0.82 (0.63, 1.07)	89/1531	80/1519	27	25	1.13 (0.83, 1.53)
DAPA-CKD	42	9/697	14/701	6.0	10	0.65 (0.28, 1.49)	8/697	19/701	4.8	11	0.42 (0.19, 0.96)
EMPA-KIDNEY	39	17/1779	11/1790	5.0	3.2	1.54 (0.72, 3.28)	30/1779	33/1790	8.8	9.6	0.92 (0.56, 1.50)
**Subtotal: NO DIABETES**	**56**	**422/7792**	**477/7813**			**0.88 (0.78, 1.01)**	**301/7792**	**303/7813**			**1.00 (0.85, 1.17)**
**TOTAL: OVERALL**	**65**	**2330/48483**	**2251/41926**			**0.86 (0.81, 0.92)**	**1434/48477**	**1338/41924**			**0.94 (0.88, 1.02)**

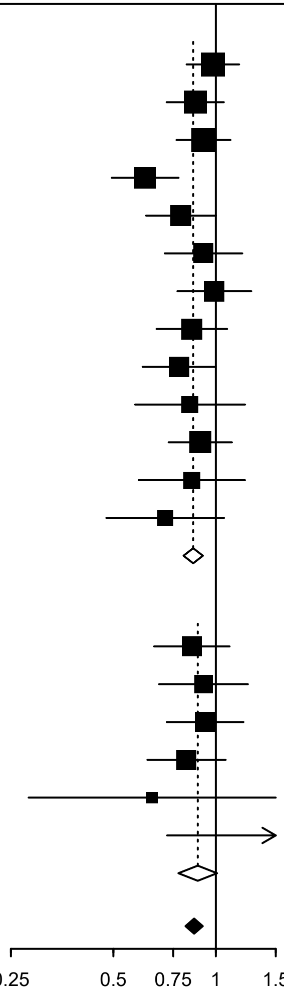

Trend across trials sorted by eGFR:
Diabetes p=0.44;
No diabetes p=0.74;

Heterogeneity by diabetes status: p=0.68

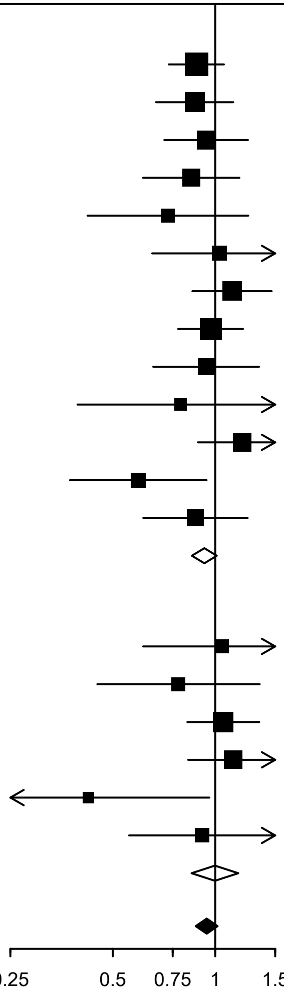

Trend across trials sorted by eGFR:
Diabetes p=0.52;
No diabetes p=0.66;

Heterogeneity by diabetes status: p=0.43

Webtable 2 provides details of when relative risks were estimated from numbers of events. CI = confidence interval. eGFR = estimated glomerular filtration rate. SGLT2i = sodium-glucose co-transporter-2 inhibitor.

Webfigure 8: Effect of SGLT2 inhibitors on KETOACIDOSIS, by diabetes status

	Average baseline eGFR (mL/min/1.73m²)	Events/participants SGLT2i	Events/participants Placebo	Rate per 1000 patient years SGLT2i	Rate per 1000 patient years Placebo	Relative risk (95% CI)	Trend across trials sorted by eGFR
**Diabetes**							
DECLARE-TIMI 58	85	27/8574	12/8569	0.7	0.3	2.18 (1.10, 4.30)	
CANVAS Program	77	13/5795	5/4347	0.6	0.3	2.33 (0.76, 7.17)	
VERTIS CV	76	19/5493	2/2745	1.2	0.2	4.75 (1.11, 20.37)	
EMPA-REG OUTCOME	74	4/4687	1/2333	0.3	0.1	1.99 (0.22, 17.80)	
DAPA-HF	63	3/1073	0/1063	1.9	0.0	5.94 (0.30, 118.53)	
EMPEROR-REDUCED	61	0/927	0/926	0.0	0.0		
EMPEROR-PRESERVED	60	4/1465	5/1471	1.2	1.5	0.80 (0.22, 2.99)	p=0.69
DELIVER	60	2/1578	0/1572	0.6	0.0	3.98 (0.18, 88.30)	
CREDENCE	56	11/2200	1/2197	2.2	0.2	10.80 (1.39, 83.65)	
SOLOIST-WHF	51	2/605	4/611	4.4	8.7	0.50 (0.09, 2.75)	
SCORED	44	30/5291	14/5286	4.3	2.0	2.14 (1.14, 4.03)	
DAPA-CKD	44	0/1453	2/1450	0.0	0.6	0.25 (0.01, 5.53)	
EMPA-KIDNEY	36	5/1525	1/1515	1.6	0.3	5.27 (0.61, 45.22)	
**Subtotal: DIABETES**	**67**	**120/40666**	**47/34085**			**2.12 (1.49, 3.04)**	
**No diabetes**							
DAPA-HF	68	0/1295	0/1305				
EMPEROR-REDUCED	63	0/936	0/937				
DELIVER	63	0/1551	0/1558				
EMPEROR-PRESERVED	62	0/1531	0/1518				
DAPA-CKD	42	0/696	0/699				
EMPA-KIDNEY	39	1/1779	0/1790				
**Subtotal: NO DIABETES**	**56**	**1/15592**					

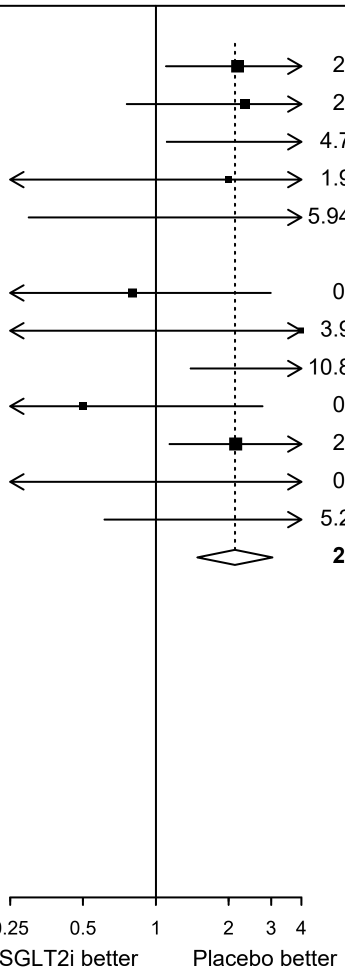

Webtable 2 provides details of when relative risks were estimated from numbers of events. CI = confidence interval. eGFR = estimated glomerular filtration rate. SGLT2i = sodium-glucose co-transporter-2 inhibitor.

Webfigure 9: Effect of SGLT2 inhibitors on LOWER LIMB AMPUTATION, by diabetes status

	Average baseline eGFR (mL/min/1.73m²)	Events/participants SGLT2i	Events/participants Placebo	Rate per 1000 patient years SGLT2i	Rate per 1000 patient years Placebo	Relative risk (95% CI)	Trend across trials sorted by eGFR
**Diabetes**							
DECLARE-TIMI 58	85	123/8574	113/8569	3.4	3.1	1.09 (0.84, 1.40)	
CANVAS Program	77	140/5790	47/4344	6.3	3.4	1.97 (1.41, 2.75)	
VERTIS CV	76	111/5493	45/2745	6.7	5.5	1.23 (0.87, 1.74)	
EMPA-REG OUTCOME	74	88/4687	43/2333	6.5	6.5	1.00 (0.70, 1.44)	
DAPA-HF	63	12/1073	9/1063	7.5	5.6	1.32 (0.56, 3.16)	
EMPEROR-REDUCED	61	12/927	9/926	9.4	7.0	1.33 (0.56, 3.15)	
EMPEROR-PRESERVED	60	15/1465	21/1471	4.7	6.5	0.72 (0.37, 1.39)	p=0.13
DELIVER	60	15/1578	21/1572	4.3	6.1	0.70 (0.36, 1.36)	
CREDENCE	56	70/2200	63/2197	12	11	1.11 (0.79, 1.56)	
SOLOIST-WHF	51	4/605	1/611	8.8	2.2	4.04 (0.45, 36.04)	
SCORED	44	32/5291	33/5286	4.5	4.7	0.97 (0.60, 1.57)	
DAPA-CKD	44	35/1453	38/1450	10	11	0.92 (0.57, 1.46)	
EMPA-KIDNEY	36	23/1525	17/1515	7.6	5.6	1.30 (0.69, 2.43)	
**Subtotal: DIABETES**	**67**	**680/40661**	**460/34082**			**1.15 (1.02, 1.30)**	
**No diabetes**							
DAPA-HF	68	1/1295	3/1305	0.5	1.5	0.34 (0.03, 3.23)	
EMPEROR-REDUCED	63	1/936	1/937	0.8	0.8	1.00 (0.06, 15.98)	
DELIVER	63	4/1551	4/1558	1.2	1.2	1.00 (0.25, 3.98)	
EMPEROR-PRESERVED	62	1/1531	2/1518	0.3	0.6	0.50 (0.04, 5.46)	p=0.23
DAPA-CKD	42	0/696	1/699	0.0	0.6	0.50 (0.02, 14.94)	
EMPA-KIDNEY	39	5/1779	2/1790	1.5	0.6	2.59 (0.50, 13.36)	
**Subtotal: NO DIABETES**	**56**	**12/7788**	**13/7807**			**0.98 (0.43, 2.25)**	
**TOTAL: OVERALL**	**65**	**692/48449**	**473/41889**			**1.15 (1.02, 1.30)**	
**TOTAL: OVERALL (excluding CANVAS)***	**64**	**552/42659**	**426/37545**			**1.06 (0.93, 1.21)**	

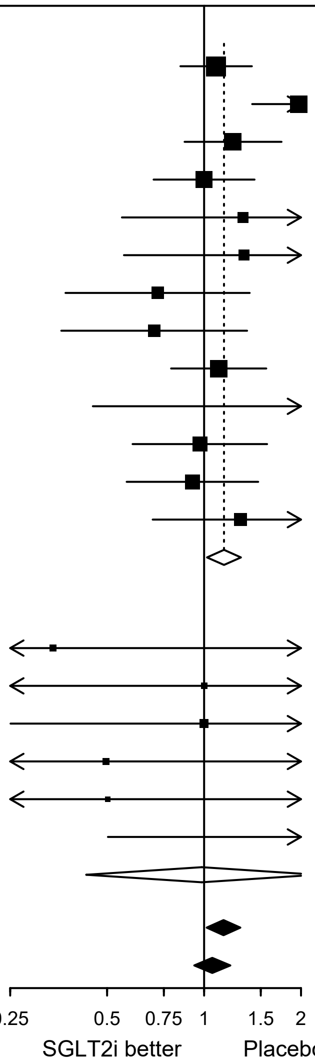

Heterogeneity by diabetes status: p=0.71

Webtable 2 provides details of when relative risks were estimated from numbers of events. *The hypothesis that SGLT2 inhibition might increase the risk of lower limb amputation was first raised by results from the CANVAS trial. The subtotal excluding CANVAS therefore reflects the combined results from the independent set of hypothesis-testing trials. CI = confidence interval. eGFR = estimated glomerular filtration rate. SGLT2i = sodium-glucose co-transporter-2 inhibitor.

Webfigure 10: Effect of SGLT2 inhibitors on ADDITIONAL SAFETY outcomes

	Mean baseline eGFR (mL/min/1.73m²)	Events/participants SGLT2i	Events/participants Placebo	Relative risk (95% CI)
**Urinary tract infections**				
High atherosclerotic CV risk trials	80	1938/24549	975/17994	1.05 (0.97, 1.13)
Stable heart failure trials	61	418/7985	358/7979	1.17 (1.02, 1.34)
Chronic kidney disease trials	44	936/12944	878/12937	1.09 (0.93, 1.27)
**TOTAL: OVERALL**	**65**	**3344/46083**	**2255/39521**	**1.08 (1.02, 1.15)**
**Serious urinary tract infections**				
High atherosclerotic CV risk trials	75	119/10180	63/5078	0.94 (0.69, 1.27)
Stable heart failure trials	61	106/7985	92/7979	1.15 (0.87, 1.52)
Chronic kidney disease trials	39	81/5453	72/5454	1.10 (0.80, 1.52)
**TOTAL: OVERALL**	**61**	**306/23618**	**227/18511**	**1.07 (0.90, 1.27)**
**Mycotic genital infections**				
High atherosclerotic CV risk trials	80	1258/24549	208/17994	3.88 (3.32, 4.53)
Stable heart failure trials	61	98/4859	34/4852	2.87 (1.95, 4.24)
Chronic kidney disease trials	44	179/12944	59/12937	2.98 (2.22, 3.99)
**TOTAL: OVERALL**	**65**	**1540/42957**	**302/36394**	**3.57 (3.14, 4.06)**
**Severe hypoglycaemia**				
High atherosclerotic CV risk trials	80	405/18754	281/13647	0.83 (0.71, 0.96)
Stable heart failure trials	62	89/10353	96/10351	0.93 (0.70, 1.23)
Chronic kidney disease trials	44	369/12944	400/12937	0.91 (0.79, 1.05)
**TOTAL: OVERALL**	**64**	**872/42656**	**779/37546**	**0.89 (0.80, 0.98)**
**Bone fracture**				
High atherosclerotic CV risk trials	80	1151/24549	811/17994	1.07 (0.98, 1.17)
Stable heart failure trials	63	228/7227	218/7220	1.04 (0.87, 1.25)
Chronic kidney disease trials	44	396/12944	377/12937	1.05 (0.91, 1.21)
**TOTAL: OVERALL**	**65**	**1787/45325**	**1415/38762**	**1.07 (0.99, 1.14)**

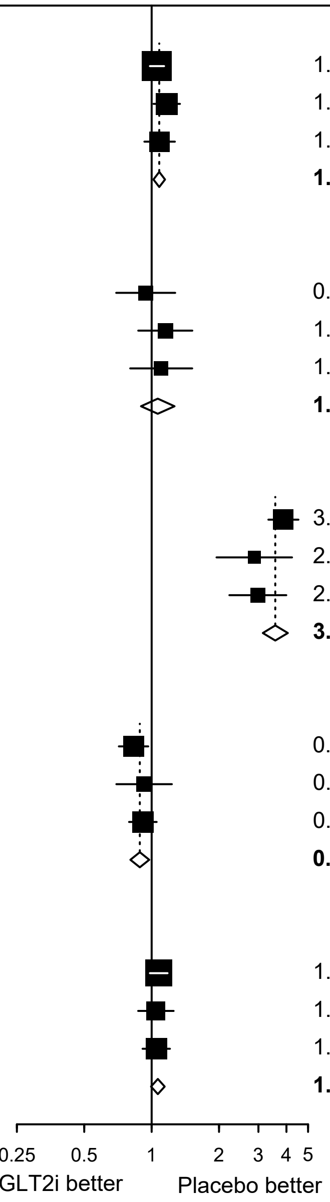

Analyses are limited to previously published reports and therefore not all outcomes are available for all trials (see Webtable 2 for detail by outcome and definition of serious urinary tract infections by trial). Data from SOLOIST-WHF included in totals but excluded from the stable heart failure trials group as it included patients with acute decompensated heart failure. There were insufficient cases of Fournier's gangrene to present a reliable estimate of risk (11 events vs 14 events in SGLT2i and placebo arms, respectively). Data extracted from previous meta-analysis (eClinicalMedicine 2021;41:101163), with additional data from DELIVER and EMPA-KIDNEY trials. CI = confidence interval. eGFR = estimated glomerular filtration rate. SGLT2i = sodium-glucose co-transporter-2 inhibitor.

LIST OF COLLABORATORS

Writing Committee

Natalie Staplin PhD*[1], Prof. Richard Haynes DM*[1,2,3], Kaitlin J. Mayne MBChB[1], Alistair J. Roddick MBBS[1,3], SMART-C collaboration (Brendon L. Neuen MBBS[2,4], Sibylle J. Hauske MD[,5,6], Prof. Stefan D. Anker MD[2,7], Prof. Martina Brueckmann MD[2,6,8], Prof. Javed Butler MD[2,9], Prof. David Z. I. Cherney PhD[2,10], Prof. Jennifer B. Green MD[2,11], Chih-Chin Liu PhD[2,12], Finnian R. McCausland MBBCh[2,13], Prof. Darren K. McGuire MD[2,14], Prof. John J. V. McMurray MD[2,15], Prof. Milton Packer MD[2,16], Prof. Vlado Perkovic MBBS[2,4], Prof. Marc S. Sabatine MD[2,17,18], Prof. Scott D. Solomon MD[2,18], Muthiah Vaduganathan MD[2,18], Prof. Christoph Wanner MD[2,19], Prof. Stephen D. Wiviott MD[2,17,18], Prof. Faiez Zannad PhD MD[2,20], Prof. Hiddo J. L. Heerspink PhD[2,4,21]), Sarah Y. A. Ng MBChB[1], Doreen Zhu MD[1], Parminder Judge PhD[1,3], David Preiss PhD[1], Prof. Martin J. Landray FMedSci[1], Prof. Colin Baigent FMedSci[1], Prof. Jonathan R. Emberson* PhD[1], William G. Herrington* FRCP[1,2,3]

SMART-C Steering Committee

Prof. Deepak L. Bhatt MD[2,18], Prof. David Z. I. Cherney PhD[2,10], Prof. Bruce Neal PhD[2,4], Brendon L. Neuen MBBS[2,4], Prof. Vlado Perkovic MBBS[2,4], Prof. Richard Haynes DM[1,2,3], William G. Herrington FRCP[1,2,3], Prof. Hiddo J. L. Heerspink PhD[2,4,21], Prof. Silvio E. Inzucchi MD[2,22], Prof. Meg J. Jardine PhD[2,4,23], Prof. Kenneth W. Mahaffey MD[2,24], Prof. Darren K. McGuire MD[2,14], Prof. John J. V. McMurray MD[2,15], Prof. Milton Packer MD[2,16], Prof. Marc S. Sabatine MD[2,17,8], Prof. Scott D. Solomon MD[2,18], Muthiah Vaduganathan MD[2,18], Prof. Christoph Wanner MD[2,19], Prof. Stephen D. Wiviott MD[2,17,18], Prof. David C. Wheeler MD[2,25], Prof. Faiez Zannad PhD MD[2,20].

* Equal contributions

Affiliations

1. Renal Studies Group, Medical Research Council Population Health Research Unit at the University of Oxford, Clinical Trial Service Unit and Epidemiological Studies Unit, Nuffield Department of Population Health (NDPH), University of Oxford, Oxford, UK.
2. SGLT-2 Meta-Analysis Cardio-Renal Trialists' Consortium (SMART-C).
3. Oxford Kidney Unit, Oxford University Hospitals NHS Foundation Trust, Oxford, UK.
4. George Institute for Global Health, Newtown, Australia; University of New South Wales, Sydney, New South Wales, Australia.
5. Vth Department of Medicine, University Medical Center Mannheim, University of Heidelberg, Heidelberg, Germany.
6. Boehringer Ingelheim International GmbH, Ingelheim, Germany.

7. Department of Cardiology (CVK), and Berlin Institute of Health Center for Regenerative Therapies, German Center for Cardiovascular Research Partner Site Berlin, Charité Universitätsmedizin, Berlin, Germany.
8. First Department of Medicine, Faculty of Medicine Mannheim, University of Heidelberg, Mannheim, Germany.
9. Baylor Scott and White Research Institute, TX and University of Mississippi, Jackson, MS, USA.
10. Department of Medicine, University of Toronto, Toronto, Ontario, Canada.
11. Division of Endocrinology, Department of Medicine and Duke Clinical Research Institute, Duke University Medical Center, Durham, NC, USA.
12. Merck & Co., Inc., Rahway, New Jersey, USA.
13. Renal Division (F.R.M.), Brigham and Women's Hospital, Boston, MA, USA.
14. University of Texas Southwestern Medical Center, Parkland Health and Hospital System, Dallas, TX, USA.
15. British Heart Foundation Cardiovascular Research Centre, University of Glasgow, Glasgow, UK.
16. Baylor Heart and Vascular Institute, Baylor University Medical Center, Dallas, TX, USA.
17. TIMI Study Group.
18. Division of Cardiovascular Medicine, Brigham and Women's Hospital, Harvard Medical School, Boston, MA, USA.
19. Würzburg University Clinic, Würzburg, Germany.
20. Université de Lorraine, Inserm, Center d'Investigations Cliniques, - Plurithématique 14–33, and Inserm U1116, CHRU, F-CRIN INI-CRCT (Cardiovascular and Renal Clinical Trialists), Nancy, France.
21. Department of Clinical Pharmacy and Pharmacology, University of Groningen, Groningen, the Netherlands.
22. Yale School of Medicine, New Haven, CT, USA.
23. NHMRC Clinical Trials Centre, University of Sydney, Australia
24. Stanford University School of Medicine, Stanford, CA 94305, USA.
25. Department of Renal Medicine, University College London, London, UK.
